# Assessment of GAFF and OPLS Force Fields for Urea: Crystal and Aqueous Solution Properties

**DOI:** 10.1021/acs.cgd.3c00785

**Published:** 2023-12-08

**Authors:** Samira Anker, David McKechnie, Paul Mulheran, Jan Sefcik, Karen Johnston

**Affiliations:** † Department of Chemical and Process Engineering, University of Strathclyde, Glasgow G1 1XJ, U.K.; ‡ Future Continuous Manufacturing and Advanced Crystallisation Research Hub, University of Strathclyde, Glasgow G1 1RD, U.K.

## Abstract

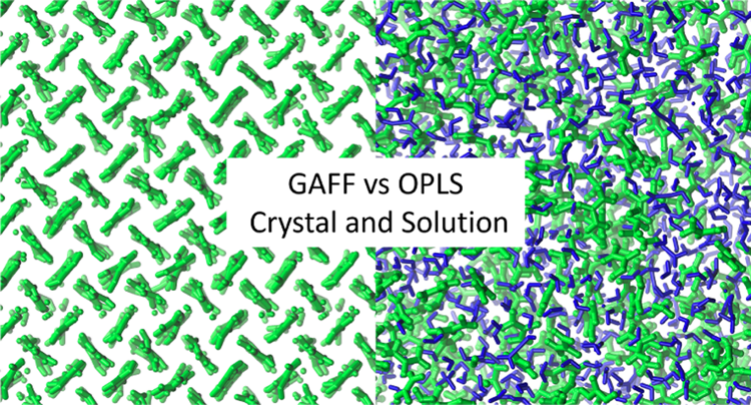

Molecular simulations such as Monte Carlo, molecular dynamics, and metadynamics have been used to provide insight into crystallization phenomena, including nucleation and crystal growth. However, these simulations depend on the force field used, which models the atomic and molecular interactions, to adequately reproduce relevant material properties for the phases involved. Two widely used force fields, the General AMBER Force Field (GAFF) and the Optimized Potential for Liquid Simulations (OPLS), including several variants, have previously been used for studying urea crystallization. In this work, we investigated how well four different versions of the GAFF force field and five different versions of the OPLS force field reproduced known urea crystal and aqueous solution properties. Two force fields were found to have the best overall performance: a specific urea charge-optimized GAFF force field and the original all-atom OPLS force field. It is recommended that a suitable testing protocol involving both solution and solid properties, such as that used in this work, is adopted for the validation of force fields used for simulations of crystallization phenomena.

## Abstract 2

Molecular dynamics simulations provide valuable insight into complex processes, such as crystallization. However, their predictions depend on the quality of the force field model. This work suggests simple tests of bulk crystal and solution properties for selecting force fields models for crystallization simulations. These tests are performed for urea using several versions of the common GAFF and OPLS force fields.

## 1 Introduction

Crystallization is an essential separation process in many applications across the food, chemical, and pharmaceutical industries. Control of crystallization is crucial in ensuring the desired quality attributes of crystalline products, such as solid form, size distribution, morphology, and purity. Molecular simulations of crystallization phenomena such as nucleation and crystal growth are a useful tool for studying crystallization since they provide an atomistic-level insight that cannot always be achieved experimentally. For instance, much work has gone into understanding the mechanisms and steps involved in the nucleation process for systems such as urea. Simulations of homogeneous nucleation of urea found that clusters arose from density fluctuations and some of these clusters became crystal nuclei.^1,2^ These nuclei had an initial crystal form that has not been observed experimentally, and only once the nuclei grew to a certain size did they spontaneously transform into the known crystal form.^1−3^ However, it is crucial to ensure that such molecular simulations adequately reproduce the properties and behaviors observed experimentally; otherwise, these studies may not provide insights relevant for the particular systems investigated.

Classical Monte Carlo, molecular dynamics (MD) and metadynamics simulations are reliant on the use of force fields to emulate material properties of the systems and phases investigated. A wide variety of force fields are available in the literature ranging from commonly used general force fields that can be applied to many different molecules to niche force fields optimizing a few parameters for one specific molecule. These force fields can also be combined in various ways; for aqueous solutions, it is particularly common to use different force fields for solute and solvent (water) molecules, but there are also examples of intermolecular parameters from one force field being paired with the intramolecular parameters of another. Regardless, the choice of the force field is important and needs to be validated for the intended application. For studies of crystallization processes, it is important for the force field to be able to reproduce both crystal and solution behaviors well. We are not aware of any standardized procedure for validating force field performance using both crystal and solution properties. Therefore, in this work, we propose a set of simple tests that can be used for crystal and solution force field validation, and we apply them to several different force fields, using urea as a model system.

Some of the most common general force fields for modeling organic molecules are Optimized Potentials for Liquid Simulations (OPLS) developed for modeling liquids and aqueous solutions of organic molecules;^4^ Assisted Model Building with Energy Refinement (AMBER) developed for modeling proteins and nucleic acids;^5^ Generalized AMBER Force Field (GAFF) developed for modeling small organic molecules and to be compatible with AMBER;^6^ Chemistry at Harvard Molecular Mechanics (CHARMM) developed for modeling proteins, nucleic acids, and lipids;^7^ and Groningen Molecular Simulation (GROMOS) developed for modeling biomolecular systems such as proteins and nucleotides.^8^ Although several of these are intended for larger molecules, it is relatively easy to apply the parameters to smaller organic molecules. Several versions are available for all of these force fields, some of which differ significantly from previous versions.

Urea is a convenient system for the study of crystal nucleation and growth since it has only one polymorph under ambient conditions and exhibits relatively fast nucleation and crystal growth. These factors, combined with its small size, also make urea well suited for molecular simulations. Hence, crystal growth and dissolution of urea have been widely studied using MD simulations.^3,9−14^

A large number of force fields are available for urea, but only a few of these have been extensively validated. An overview of the force fields that have been tested for urea in one way or another is given in Table 1. Out of the force fields that have been used to model urea crystals and solutions only OPLS and GAFF have been widely used. Only the solution phase has been tested for OPLS, and for GAFF only crystal phase tests have been performed; however, subsequent studies involve both the crystal and solution phases, which implies that both the OPLS and GAFF force fields can reliably reproduce both phases to some extent.

**Table 1:** Overview of Force Fields That Have Been Tested for Urea^a^

urea model	water model	application	tested properties
OPLS*^15^ (urea-specific)	TIP4P	solution	absolute free energy of hydration, solution structural correlations^15^
OPLS*^15^ (urea-specific)	TIP3P	solution	density, diffusion coefficients^16^
CHARMM^7^	TIP3P	dimers and solution	diffusion coefficients, solution structural correlations^17^
CHARMM^7^	TIP3P	crystal and solution	solution structural correlations, diffusion and solvation free energy; bulk crystal density and enthalpy of sublimation; solubility^18^
OPLS*^15^ + CHARMM^19^	SPC/E	solution and cosolvent	density, solution structural correlations, diffusion, and dielectric properties^20^
OPLS*^15^ + GROMOS^21,22^	SPC	solution	density, energy of solution, heat of solvation, free enthalpy of desolvation, and urea diffusion^23^
GAFF^6^	TIP3P	crystal and solution	bulk crystal crystal lattice parameters and melting point temperature (from a solid–liquid interface)^11^
GAFF^24^ (urea-optimized)	N/A	dimers and crystal	cohesive energy, sublimation, and melting point temperatures^25^
KBFF^26^ (Kirkwood-Buff, urea-specific)	SPC/E, SPC, TIP3P	solution	solution structural correlations, partial molar volumes, isothermal compressibility, activity derivatives and coefficients, density, relative permittivity, diffusion constant, and crystal lattice parameters^26^
KBFF^26^ (urea-specific)	TIP3P	solution	density, diffusion coefficients^16^
SAPT-FF^18^ (polarized)	SWM4-NDP (polarized)	crystal and solution	solution structural correlations, diffusion and solvation free energy; bulk crystal density and enthalpy of sublimation; solubility^18^
COMPASS^27^ (polarized)	N/A	crystal	crystal lattice parameters^27^
AMOEBA^28,29^ (polarized)	N/A	crystal	crystal lattice parameters^30^

a *The urea-specific OPLS^15^ force field only has intermolecular parameters; therefore, simulations have either been done without intramolecular interactions or by taking these parameters from the secondary force field listed after the + sign.

The OPLS-GROMOS force field was used in the earlier simulations of urea crystal growth and dissolution.^9,10^ However, the GAFF force field has been favored in more recent studies, due to the broad range of other molecules that can also be modeled with GAFF.^11^ The GAFF force field has been used to study the effects of additives^11^ and solvents^12,31^ on urea crystallization and to simulate homogeneous nucleation using well-tempered metadynamics with enhanced sampling.^1,2,31^ The dissolution of small nuclei-like crystals has been studied using both the GAFF^3^ and urea-optimized GAFF^14^ force fields.

The aim of this paper is to outline a series of general tests that can be used for the validation of force fields for crystallization studies by considering both the crystal and solution properties predicted using these force fields. A range of OPLS and GAFF force fields that have been used to study urea will be considered. This will enable the most suitable force field to be identified for future work on urea crystal nucleation and growth. Further development and improvement of the force fields will enable MD simulations to be used for more insightful studies of crystallization phenomena.

## 2 Methodology

In this section, we give an overview of the nine selected GAFF and OPLS force fields tested for urea, summarize the molecular dynamics simulations, and describe how the crystal and solution systems were set up.

### 2.1 Selection of Force Fields

The original GAFF force field,^6,32^ denoted here as GAFF1, was developed for use with most organic and pharmaceutical molecules. There have been some updates to the GAFF1 parameters and a second generation, GAFF2, has been developed where GAFF2 includes both updated bonded and nonbonded parameters compared to GAFF1.^6,32,33^ There are no charges directly associated with GAFF, and these need to be calculated on a molecule by molecule basis. The Antechamber tool,^32^ used to obtain the force field parameters, includes a default option for calculating charges based on AM1-BCC charge model, which does not require any further inputs. These two force fields are referred to as GAFF1 (version 1.81, AM1-BCC charges^6,32^) and GAFF2 (version 2.11, AM1-BCC charges^6,32^) in the rest of this paper.

An alternative version of GAFF was specifically developed for urea.^24^ This version geometrically optimized the bonded potential parameters but did not alter the nonbonded Lennard-Jones parameters from version 1. Here, the RESP charge model was used, and seven sets of charges were calculated for different orientations of urea dimers (D1-D7), and from these, D1 and D3 were chosen for this work, since D1 was based on the crystal structure and D3 was recommended as the most suitable overall. These two force fields are referred to as GAFF-D1 (optimized GAFF1, RESP-D1 charges^24^) and GAFF-D3 (optimized GAFF1, RESP-D3 charges^24^).

The OPLS force field was developed as a series of intermolecular parameters for different types of organic molecules. There were no associated intramolecular parameters; the molecules were simply kept rigid throughout the simulations, with the structure being based on experimental parameters. The original OPLS force fields were not all-atom force fields but included united-atom terms for carbon atoms where all hydrogen atoms bonded to carbon atoms were implicitly included in the carbon atom parametrization. Versions were developed for liquid hydrocarbons,^34^ peptides and amides (OPLS-Amide),^35,36^ liquid alcohols,^37^ proteins,^38,39^ and nucleotide bases.^40^ The general all-atom OPLS force field (OPLS-AA) was developed for both liquid and solid simulations^4,41^ and we have chosen to test this as it is widely used. OPLS-AA consists of the bond and angle parameters from AMBER,^5,42^ newly calculated dihedral and improper parameters,^4^ and OPLS intermolecular parameters. The parameters for OPLS-AA were obtained from tables in publications by Jorgensen et al.^4^ and Weiner et al.^5^ The LigParGen software has been created, by the developers of OPLS, to more easily obtain the OPLS force field parameters from an input structure file.^43−45^ However, when tested, LigParGen produced different parameters compared to OPLS-AA, and these are also tested and denoted as OPLS-AA-N. OPLS-AA and OPLS-AA-N differ by the charges, Lennard-Jones parameters of the carbon atom, and OPLS-AA-N has one additional angle parameter. Prior to the parametrization of OPLS-AA, a urea-specific version was developed^15^ (OPLS-Urea), based on OPLS-Amide,^35,36^ versions of this continue to be used. We also tested the OPLS-AA as above but with the intermolecular parameters of OPLS-Urea, called OPLS-AA-D.

OPLS-Urea has only intermolecular parameters, this has been used as is for solution simulation^16,46^ and has been combined with intramolecular parameters from other force fields, including CHARMM22^20^ and GROMOS96,^23^ both of which were only used for solutions. The combination with GROMOS96, which was implemented with rigid bond lengths, has been extensively validated by Smith et al.,^23^ with comparisons of density, enthalpy of mixing, free enthalpy of urea hydration, and urea diffusivity properties to experimental data. This led to the subsequent use of this force field by Piana et al.^9,10^ in their work on crystal growth and dissolution. Therefore, we also test this force field, referring to it as OPLS-S, but implement it without rigid bonds. We note that there is some discrepancy between the dihedral parameters in the GROMOS96 source^8^ and those cited by Smith et al.^23^ We have not been able to access the manual^21^ used by Smith et al.,^23^ however, the GROMOS 53A5 and 53A6 parameter set^8^ was identified as being the relevant parameter set as it the first published set which contains the parameters used by Smith et al.^23^ First, the O–C–N–H dihedrals are applied to only two out of the four instances of these dihedrals, without any explanation of this choice. In addition, the parameters chosen are taken from the X–C–C–X (6-ring) example and not the X–C–N–X example. Therefore, we also tested a version that uses the original GROMOS96 dihedrals (OPLS-G).

A summary of the urea force fields investigated in this work is given in Table 2.

**Table 2:** Nine Selected Force Fields, Summarizing the Source of Bonded, Lennard-Jones, and Electrostatic Parameters

force field	bonded	Lennard-Jones	electrostatics
GAFF1	GAFF1^6,32^	GAFF1^6,32^	calculated AM1-BCC^32^
GAFF2	GAFF2^6,32^	GAFF2^6,32^	calculated AM1-BCC^32^
GAFF-D1	optimized GAFF1^24^	GAFF1^6,24^	optimized RESP^24^
GAFF-D3	optimized GAFF1^24^	GAFF1^6,24^	optimized RESP^24^
OPLS-AA	AMBER^5,42^ and OPLS-AA^4^	OPLS-AA^4^	OPLS-AA^4^
OPLS-AA-N	OPLS-AA-N^4,43^	OPLS-AA-N^4,43^	OPLS-AA-N^4,43^
OPLS-AA-D	OPLS-AA-N^4,43^	OPLS-Urea^15^	OPLS-Urea^15^
OPLS-S	GROMOS based^8,23^	OPLS-Urea^15,23^	OPLS-Urea^15,23^
OPLS-G	GROMOS^8^	OPLS-Urea^15,23^	OPLS-Urea^15,23^

A variety of water force fields have been used in combination with various urea force fields including SPC/E,^3,14^ SPC,^9,10,14,23,47^ TIP3P,^1,11−15^ and TIP4*P*/2005.^14^ GAFF was developed with TIP3P water and OPLS with TIP4P water; however, both are compatible with and have been successfully used with most of these water force fields. While all models reproduce the density of pure water well, SPC/E is best for reproducing bulk dynamics and structures, including self-diffusion coefficients, followed by TIP4P, SPC, and TIP3P.^48−50^ The SPC/E model^49,51^ was chosen for this work due to its ability to reproduce pure water properties well and its good performance in previous works with other small organic molecules (modeled with several different force fields including GAFF and OPLS).^50,52^

### 2.2 MD Simulation Details

Molecular dynamics simulations were performed using the LAMMPS software.^53,54^ LAMMPS input files are available as Supporting Information. All simulations were performed in the isothermal–isobaric ensemble (NPT) using a time step of 2.0 fs, with thermodynamic and structural properties sampled every 2000 fs. The temperature and pressure were controlled by a Nosé–Hoover thermostat and barostat. Damping parameters of 0.2 and 2.0 ps were used for the temperature and pressure, respectively. The size of the simulation cell is rescaled independently for each of the three axes; however, the cell angles are constrained to the initial value of 90°. Periodic boundary conditions were applied in all dimensions for both the crystal and the solution simulations.

A cutoff of 9.0 Å was used for both Lennard-Jones and short-range electrostatic interactions, which is the default value used with both GAFF and OPLS. Long-range electrostatics were calculated using a particle–particle–particle–mesh with a relative error in forces of 1 × 10^–4^. Long-range Lennard-Jones interactions and their effects on energy and pressure are corrected for using eq 5 of Sun.^55^

The strength of Lennard-Jones interactions between 1–4 bonded atoms was set to 0.5 of the full interaction strength and set to 0 for 1–2 and 1–3 bonds. The strength of electrostatic interactions between 1–4 bonded atoms was set to 0.83333333 and 0.5 of the full interaction strength for the GAFF and OPLS force fields, respectively, and set to 0 for 1–2 and 1–3 bonds. These scale factors were set according to the defaults of GAFF^6^ and OPLS.^4,23^

### 2.3 Crystal Setup and Analysis

**Figure 1:**
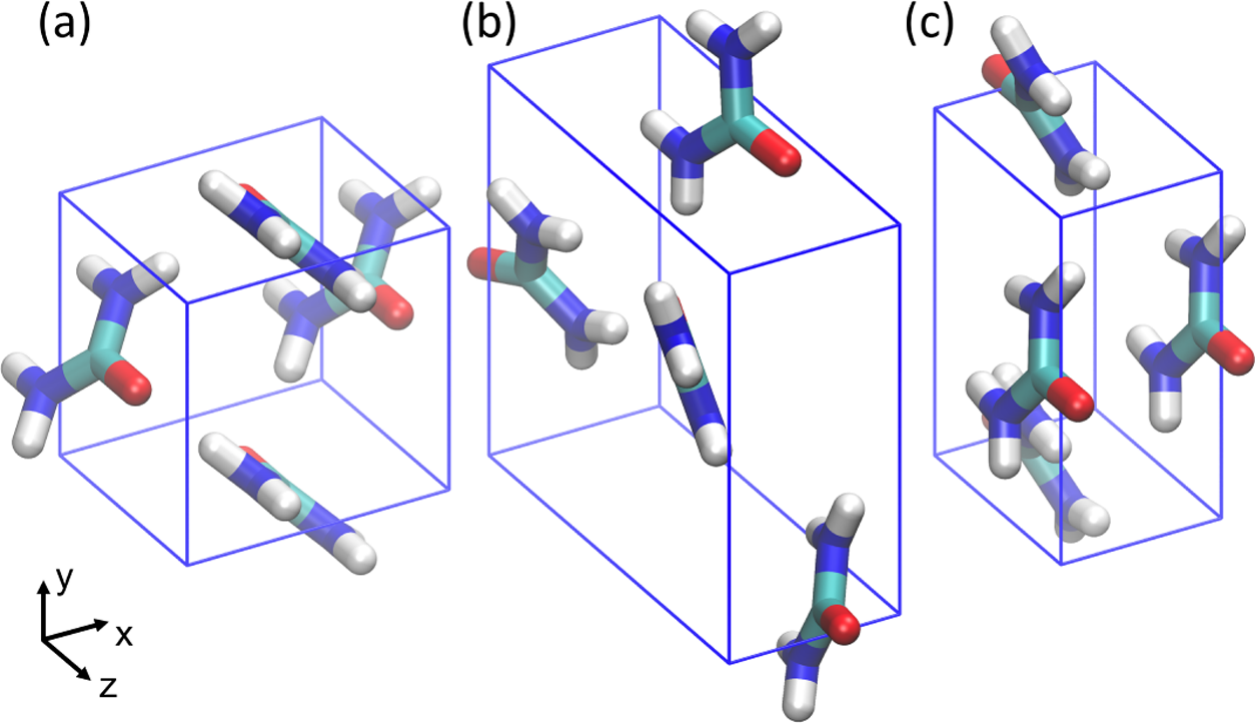
Urea unit cells for (a) form I, (b) form III, and (c) form IV. Form I and IV have only two molecules in the unit cell; four are shown here to give a better visualization of the packing structure.

There are four known crystal structures of urea that have been observed experimentally, denoted here as forms I, III, IV, and V, where only form I exists at ambient conditions. Lattice parameters are only available for forms I, III, and IV, and these three structures are shown in Figure 1. Form I and IV have a similar structure, with the same number of NH···O hydrogen bonds, the structure of form III differs more and forms one less NH···O bond.^56^

Form I has space group P42_1_m (no. 113), which is a tetragonal structure with lattice parameters *a* = *b* ≠ *c* and α = β = γ = 90°,^57^ as seen in Figure 1a. The form I unit cell is composed of two urea molecules that are perpendicular to each other, when viewed along the z direction. A form II structure was initially discovered, but it has not been observed since and it is thought that form II corresponds to form IV.^56,58^ Form III has the space group *P*2_1_2_1_2_1_ (no. 19), which is an orthorhombic structure with lattice parameters *a* ≠ *b* ≠ *c* and α = β = γ = 90°, as seen in Figure 1b. The form III unit cell is composed of four urea molecules, this is also a high-pressure form, observed experimentally above 0.48 GPa.^59^ Form IV has space group *P*2_1_2_1_2 (no. 18), which is also an orthorhombic structure with lattice parameters *a* ≠ *b* ≠ *c* and α = β = γ = 90°, this structure is a high-pressure form, observed experimentally above 2.80 GPa,^59^ as seen in Figure 1c. The form IV unit cell is also composed of two urea molecules; it is similar to form I, except that the molecules are aligned in a herringbone pattern along the *z* direction instead of being perpendicular to each other, and the overall structure is more compressed. Form V is only observed at pressures above 7.8 GPa, and no lattice parameters have been obtained for this structure.^58−60^

Urea crystals were set up in the form I and form IV unit cells. Form I was selected as it is the ambient form. Form III was not considered since it is a high-pressure form, and we are interested only in crystallization at ambient conditions. Despite the high pressure required to obtain form IV experimentally, this form was considered due to the similarities between form IV and distorted forms seen in some of the form I simulations. For each force field, the unit cell was energy-minimized and the optimized unit cell was used to build a crystal supercell of 5 × 5 × 5 unit cells, which was also energy-minimized. The energy minimization was performed with the Polak-Ribiere version of the conjugate gradient algorithm. The size and shape of the simulation box were allowed to vary independently in all dimensions during the minimization with a maximum allowed fractional volume change of 0.0001 per iteration. NPT simulations were performed with an anisotropic barostat which allowed the crystal to independently change its *a*, *b*, and *c* lattice parameters. The NPT simulations were performed at temperatures of 300, 400, 450, and 500 K, all at 1 atm, and for 10 ns.

The cohesive energy (*E*_cohesive_) was calculated as follows
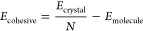
where *E*_crystal_ is the potential energy of the crystal, *N* is the number of molecules in the bulk crystal, and *E*_molecule_ is the potential energy of one molecule in vacuum for the same conditions. Reference simulations of one urea molecule in vacuum were performed for each force field; these were run for 1 ns, and the potential energy over this period was averaged.

### 2.4 Solution Setup and Analysis

Ten different concentrations of urea solutions were tested for each urea force field as well as a single simulation with pure water. The solutions contained 1000 water molecules and an increasing number of urea molecules from 0 to 1000, with corresponding concentrations in g_urea_ kg_water_^–1^ (referred to from here onward as g kg^–1^) and percentage mass of urea (referred to from here onward as %mass) given in Table 3. The solution concentrations range from very dilute at 33.34 g kg^–1^ (3.23% mass) to highly supersaturated at 3334 g kg^–1^ (76.9% mass), where the experimental solubility is 1200 g kg^–1^ (54.5% mass) at 300 K.^61^

**Table 3:** Ten Selected Solution Compositions

molecules		mass (Da)		concentration	
urea	water	urea	water	g kg^–1^	% mass
0	1000	0.00	18016	0.00	0.00
10	1000	600.62	18016	33.34	3.23
50	1000	3003.1	18016	166.7	14.29
150	1000	9009.3	18016	500.1	33.34
200	1000	12012	18016	666.8	40.00
300	1000	18018	18016	1000	50.00
400	1000	24024	18016	1334	57.15
500	1000	30031	18016	1667	62.50
600	1000	36037	18016	2000	66.67
1000	1000	60062	18016	3334	76.93

The systems were set up by random insertion of the urea molecules into a simulation box, followed by random insertion of the water molecules. An energy minimization was performed to ensure that there were no overlapping atoms or molecules. The energy minimization was performed using a steepest decent algorithm with 0.0001 stopping tolerances for both the energy and forces. NPT simulations were performed at 1 atm and 300 K, using an isotropic barostat. Simulations were equilibrated for 2 ns, followed by a 20 ns production run. The SHAKE algorithm was used to keep the bond lengths and angles fixed in the SPC/E water model.

The mean square displacement (MSD) of urea was calculated as follows
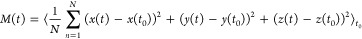
using data sampled every 10 ps and is averaged over the number urea molecules, *N*. Here, the MSD is denoted as *M*(*t*); *x*(*t*), *y*(*t*), and *z*(*t*) are the coordinates of the center of mass of the urea molecule at time step *t*; and *x*(*t*_0_), *y*(*t*_0_), and *z*(*t*_0_) are the initial positions. Multiple time origin MSDs, also known as windowed MSDs, are used to maximize the use of the available data, by also using all but the last *t* value as *t*_0_ values.

The diffusion coefficient, *D*, was calculated using the Einstein equation
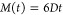
using the gradient of the M(t) in the time interval between 1 and 10 ns.

### 2.5 Force Field Validation Protocol

The bulk crystal structure, for each relevant polymorph, is simulated at ambient conditions, and the following properties are tested:

- Crystal lattice parameters
- Crystal density
- Cohesive energy

Additional bulk crystal simulations are performed at higher temperatures to obtain insight into the crystal stability and melting behavior.

Aqueous solutions are simulated for both undersaturated and supersaturated concentrations, and the following properties are tested:

- Solution density
- Solution radial distribution coefficients
- Diffusion coefficient of urea

## 3 Results and Discussion

In this section, we first present the results of the bulk crystal simulations and then present studies of the aqueous solutions.

### 3.1 Crystal Properties

In this section, we investigate the bulk urea crystal properties under ambient conditions. We present the crystal lattice parameters, densities, and cohesive energies at 300 K. We also present the crystal lattice and densities at temperatures of 400, 450, and 500 K, along with a discussion of how the higher temperatures affect the crystal form favored by particular force fields.

#### 3.1.1 Crystal Structure

The lattice parameters of the energy-minimized form I structure for each force field are shown in Table 4. The experimentally measured lattice parameters at 12 K are also shown.^57^ All force fields slightly underestimate *a* and overestimate *c*, and the density, ρ, is overestimated by all of the force fields. OPLS-S and OPLS-G are relatively close to the experimental values, overestimating the density by less than 1%, the worst performing force field GAFF-D1 overestimated this by 11%. In general, energy-minimized lattice parameters from the OPLS force fields are in better agreement with experiment than those obtained using the GAFF force fields.

**Table 4:** Lattice Parameters in Å and Density, ρ, in g cm^–3^ for the Form I Crystal Structure after Energy Minimization^a^

force field	*a* = *b* (Å)	*c* (Å)	ρ (g cm^–3^)
expt.^57^	5.565	4.684	1.375
GAFF1	5.324	4.820	1.460
GAFF2	5.321	4.774	1.476
GAFF-D1	5.221	4.810	1.521
GAFF-D3	5.328	4.811	1.460
OPLS-AA	5.412	4.795	1.420
OPLS-AA-N	5.350	4.830	1.442
OPLS-AA-D	5.415	4.786	1.421
OPLS-S	5.493	4.785	1.382
OPLS-G	5.490	4.775	1.386

a Experimental values were measured at 12 K and ambient pressure.

**Figure 2:**
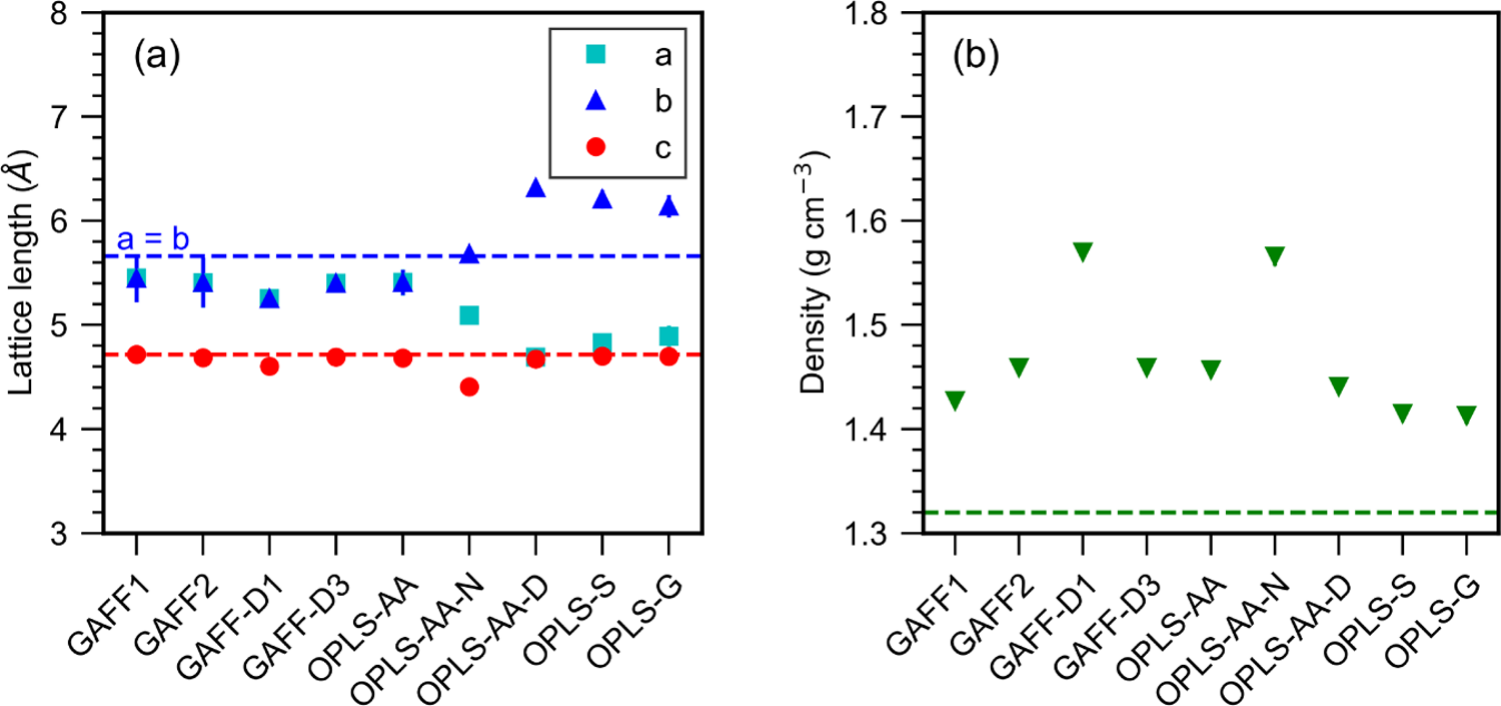
Average (a) lattice parameters and (b) density for form I urea crystal at 300 K. Error bars, representing the standard deviation, are in most cases smaller than the symbols. Tabulated values with the standard deviation are given in the Supporting Information. Horizontal lines represent experimental values at 301 K.^62^

**Figure 3:**
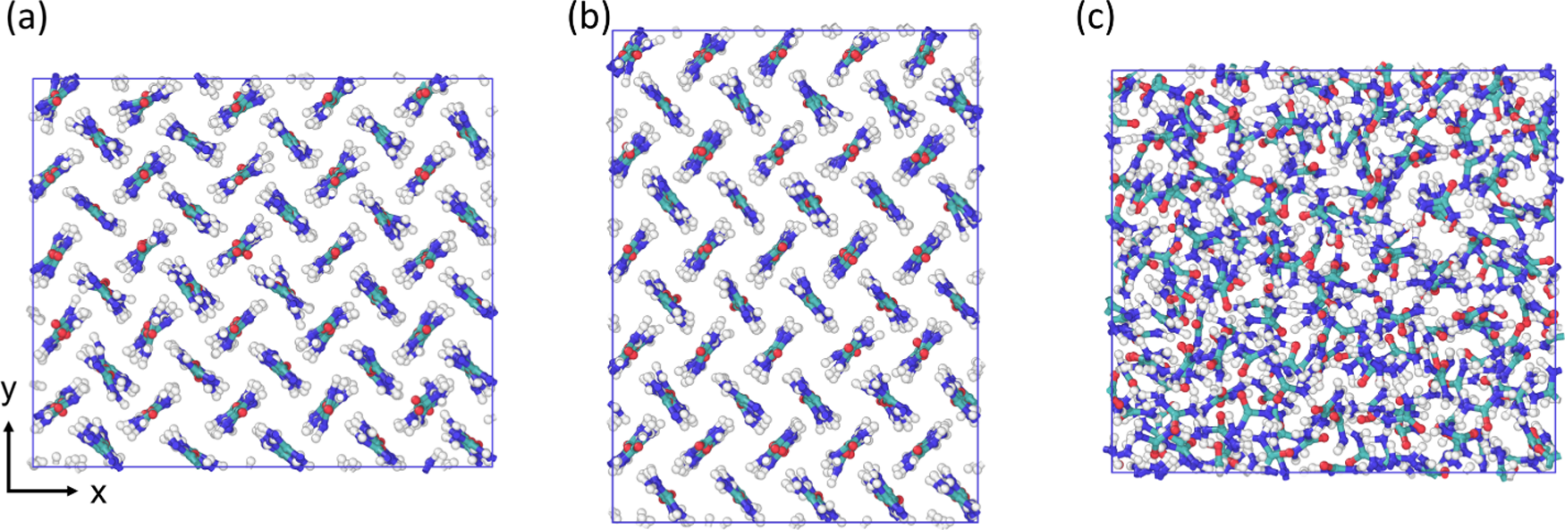
Snapshots of the crystal structure at the end of the 10 ns simulation at 300 K, (a) form I (GAFF1 representative of GAFF2, GAFF-D1, GAFF-D3, and OPLS-AA), (b) distorted form I/IV (OPLS-AA-D representative of OPLS-S and OPLS-G), and (c) amorphous (OPLS-AA-N).

We also investigated the variation of the crystal structure at 300 K and the average lattice parameters are shown in Figure 2, and exemplar crystal structures are shown in Figure 3. Tabulated values of our results with the standard deviation are given in the Supporting Information. All systems started with the 5 × 5 × 5 supercell corresponding to the energy-minimized form I structure. All four GAFF force fields and OPLS-AA retained the form I structure at 300 K, as shown in Figure 3a. The *a* and *b* lattice parameters fluctuated around an average value where *a* = *b*. The fluctuations were greatest for GAFF1 and GAFF2 where the standard deviation of the fluctuations was 4% of the mean *a* and *b* values; this was only 2% for OPLS-AA, 1.5% for GAFF-D3, and 0.6% for GAFF-D1. These fluctuations could be an effect of the Nosé–Hoover barostat and thermostat.

For OPLS-S, OPLS-G, and OPLS-AA-D, a different crystal structure was obtained, with the lattice parameters *a* < *b*, as shown in Figure 3b. Despite the changes in the *a* and *b* lengths, the molecules in the distorted form retain their perpendicular alignment to each other so that this structure is similar to form I. The nonequal *a* and *b* lengths are similar to form IV; however, in form IV, the molecules are not aligned perpendicularly. Therefore, this distorted structure can be seen as being similar to both forms I and IV. Similar distortions have been reported by Piana and Gale,^9^ in their work on urea crystal dissolution and growth, using the OPLS-S force field and by Weerasinghe and Smith^26^ in the development of the urea KBFF force field. OPLS-AA-N does not retain the form I crystal structure and instead becomes amorphous with *a* ≠ *b* ≠ *c* and only very small fluctuations in these parameters, as shown in Figure 3c.

The snapshot shown in Figure 3a is simply the configuration of GAFF1 at the end of the simulation, where the *a*: *b* ratio is approximately opposite to that of Figure 3b. The two structures are significantly different since the structure in (a) changes shape continually whereas the structure in (b) does not. The shape in (a) fluctuates between *a* = *b*, *a* > b, *a* = *b*, *a* < *b*... with an average value of *a* = *b*, whereas the fluctuations to the shape in (b) always maintain *a* < *b*.

All of the force fields overestimate the density significantly compared to the experimental crystal density of 1.33 g cm^–3^.^61^ For form I the density performance is best for OPLS-G and OPLS-S with densities of 1.413 ± 0.008 and 1.415 ± 0.007 g cm^–3^, and worst for GAFF-D1 and OPLS-AA-D with densities of 1.570 ± 0.007 and 1.566 ± 0.008 g cm^–3^.

Similar performance was obtained in other simulations of urea crystals. Density values of ∼1.382 g cm^–3^ (Salvalaglio et al.^11^ density calculated from reported lattice parameters) and later ∼1.46 g cm^–3^ (Francia et al.^63^) were obtained using GAFF1. These two values were obtained from two different studies within the same research group, showing the effect differences in application have on the results obtained with the same force field. Simulations with other force fields have obtained values of 1.30 g cm^–3^ (Jeong et al.^18^) using a specially developed polarizable force field, 1.38 g cm^–3^ (Jeong et al.^18^) with CHARMM, and ∼1.512 g cm^–3^ (Weerasinghe and Smith^26^ density calculated from reported lattice parameters) with KBFF (at a reduced temperature of 123 K). Our results fit well within the range of previously obtained results, highlighting the importance of both the force field and simulation conditions on the results obtained.

This higher density obtained in the current studies is an indication that the force field intermolecular interactions are too strong. For example, there are various parameter differences between GAFF1, GAFF-D1 and GAFF-D3, the most significant difference between these is in the partial charges. For these three force fields, the partial charges are lowest for GAFF1 and highest for GAFF-D1, corresponding to the ranking of the crystal densities. Similarly, for the OPLS force fields, OPLS-AA, OPLS-AA-N, and OPLS-AA-D have very similar parameters with different partial charges. OPLS-AA-N has some very strong charges, which result in a very dense solid (the crystal structure is lost). Comparing OPLS-AA to OPLS-AA-D, OPLS-AA has slightly larger partial charges and correspondingly a larger crystal density.

Due to the observed distortion in form I, we also studied the properties of the form IV structure. The lattice parameters and density of the energy-minimized form IV unit cell are shown in Table 5. The experimentally measured lattice parameters at 296 K and 2.96 GPa are also shown,^59^ although we note that the energy-minimized structure would correspond to a 0 K, low pressure condition. However, there are only two reported form IV structures^56,59^ on the Cambridge Structural Database, so there is not sufficient data available to extrapolate this to 0 K or 1 atm.

**Table 5:** Lattice Parameters in Å and Density, ρ, in g cm^–3^ for Form IV Crystal Structure after Energy Minimization^a^

force field	*a* (Å)	*b* (Å)	*c* (Å)	ρ (g cm^–3^)
expt.^59^	3.408	7.362	4.648	1.711
GAFF1	3.505	7.523	4.847	1.561
GAFF2	3.499	7.102	4.796	1.673
GAFF-D1	3.512	7.052	4.813	1.673
GAFF-D3	3.507	7.454	4.817	1.584
OPLS-AA	3.576	7.614	4.883	1.500
OPLS-AA-N	3.646	7.181	5.222	1.459
OPLS-AA-D	3.698	7.569	4.882	1.460
OPLS-S	3.660	7.688	4.886	1.451
OPLS-G	3.649	7.696	4.888	1.453

a Experimental values are presented at 296 K and 2.96 GPa.

All of the force fields overestimate *a* and *c. b* is underestimated by GAFF1, GAFF-D1, and OPLS-AA-N and overestimated by the remaining force fields. The density, ρ, is underestimated by all of the force fields, but GAFF2 and GAFF-D1 are relatively close to the experimental values, underestimating the density by less than 3%, OPLS-AA underestimates it by 12% and the remaining OPLS force fields performed equally badly underestimating this by 15%. In general, the energy-minimized lattice parameters from the GAFF force fields are in better agreement with experiment than those obtained using the OPLS force fields. This is opposite to the behavior obtained for form I.

**Figure 4:**
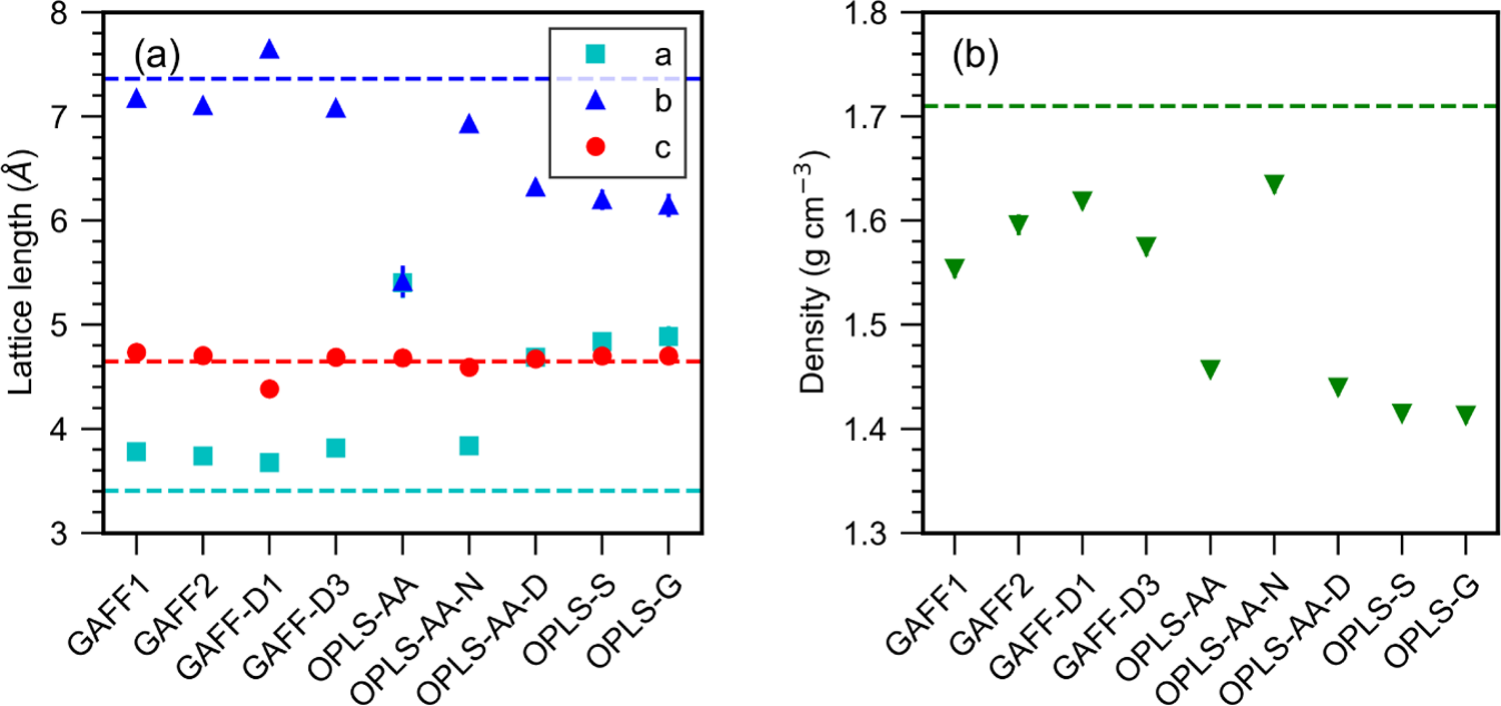
Average (a) lattice parameters and (b) density for form IV urea crystal at 300 K. Error bars, representing the standard deviation, are in most cases smaller than the symbols. Tabulated values with the standard deviation are given in the Supporting Information. Horizontal lines represent experimental values at 296 K and 2.96 GPa.^59^

**Figure 5:**
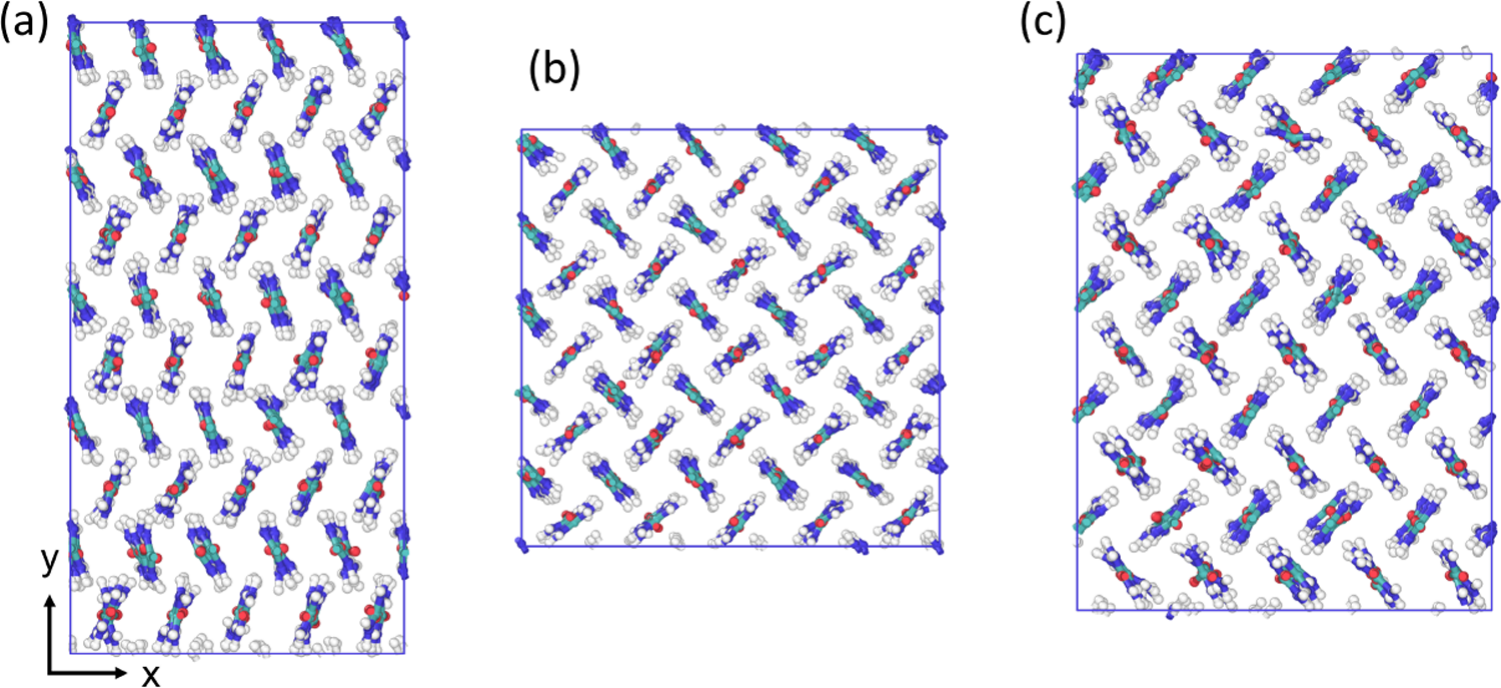
Snapshots of the crystal structure at the end of the 10 ns simulation at 300 K, (a) form IV (GAFF1 representative of GAFF2, GAFF-D1, and GAFF-D3), (b) form I OPLS-AA, and (c) distorted form I/IV (OPLS-AA-D representative of OPLS-S and OPLS-G).

We also investigated the variation of the form IV crystal structure using the 5 × 5 × 5 supercell at 300 K. The average lattice parameters and density are shown in Figure 4, with exemplar crystal structures shown in Figure 5. Tabulated values of our results with the standard deviation are given in the Supporting Information.

**Figure 6:**
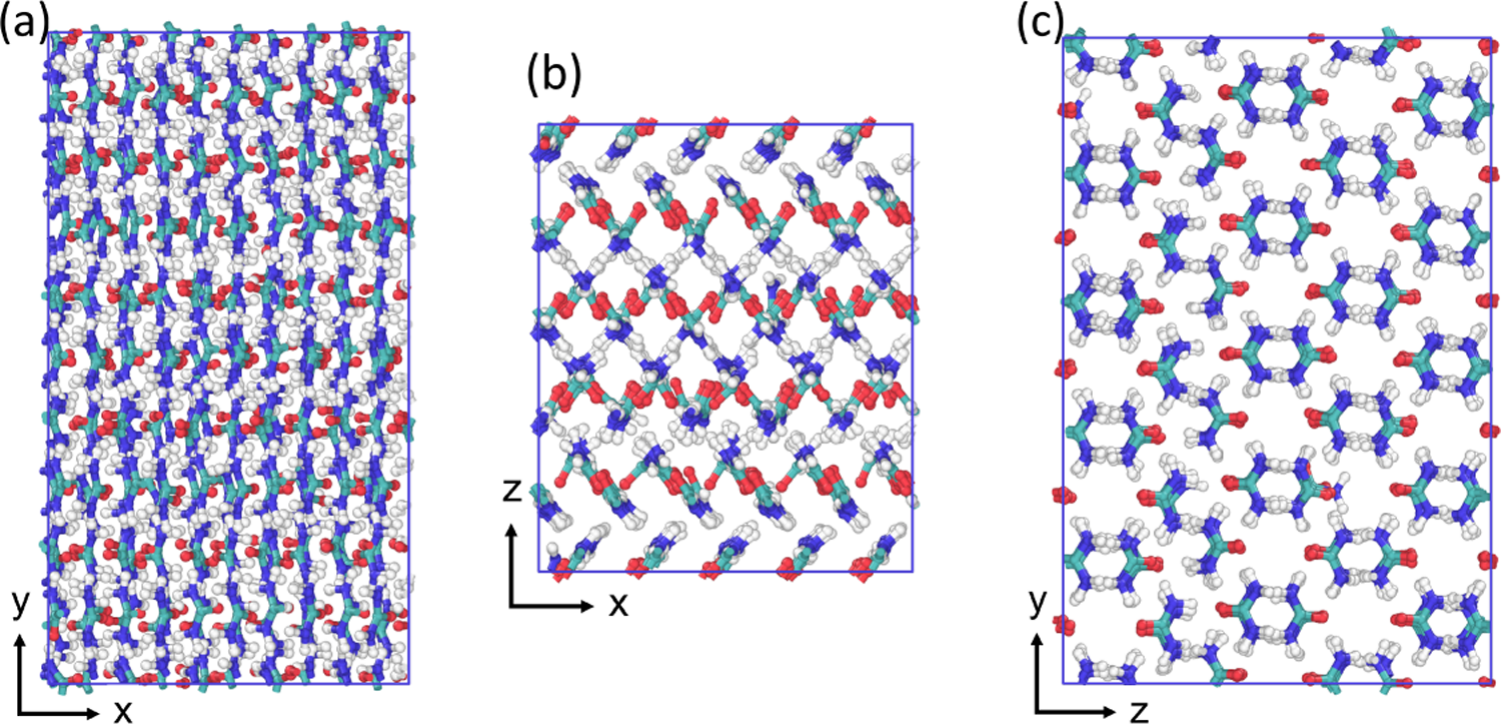
Snapshots of the new crystal structure attained by OPLS-AA-N at the end of the 10 ns simulation at 300 K viewed along the (a) *X*–*Y* plane, (b) *X*–*Z* plane, and (c) *Z*–*Y* plane.

All of the GAFF force fields retained the form IV structure at 300 K as shown in Figure 5a. The fluctuations in the lattice parameters were much less than for the form I structure. The standard deviation in *a* and *b* is seven times smaller for form IV than form I for GAFF1 and GAFF2, for GAFF-D3 it is three times smaller, and it is unchanged for GAFF-D1. The standard deviation in the *c* length is much less significant for all of the force fields in both forms I and IV. OPLS-AA reverted to the form I structure, as shown in Figure 5b, indicating that this force field is the most stable in the form I structure. The lattice parameters for OPLS-AA-D, OPLS-S, and OPLS-G differ from the experimental form IV structure but instead take on the distorted structure also obtained from the form I simulations using these force fields; this structure is shown in Figure 3b and also in Figure 5c, starting from forms I and IV, respectively. Despite the lattice parameters being similar to the experimental values, the OPLS-AA-N force field does not retain the form IV structure, instead adopting a different crystal form, as shown in Figure 6. This new form may be one of the new structures found by the recent work carried out to predict the polymorphs of urea.^63,64^

For form IV, a comparison is made to the experimentally measured lattice parameters and the corresponding density calculated from this. This is unlikely to compare directly to the values obtained from the simulations since there is a significant pressure difference between these two. The simulations were done at 1 atm, whereas the experiments are done above 2.8 GPa (∼27,000 atm). However, since the IV form is seen in the simulations, comparison to measured parameters is relevant.

#### 3.1.2 Cohesive Energy

Table 6 shows the cohesive energy of forms I and IV with the various force fields averaged over the NPT simulation. Tabulated values of the crystal potential energy are given in the Supporting Information. For all of the GAFF force fields, form IV is the lower energy structure, albeit with only a slight difference between the two forms. This indicates that form IV is more stable than form I for these force fields. This is consistent with the findings of Francia et al.,^63^ who used Crystal Structure Prediction methods alongside molecular dynamics using GAFF1 to study the relative energy rankings of different polymorphs of urea. OPLS-AA-N has a significant difference in cohesive energy between the form I simulations (which was an amorphous solid) and the new crystal structure found from the form IV simulation. There was no difference in cohesive energy for the remaining OPLS force fields, which is expected since they reverted to the same crystal structures.

**Table 6:** Cohesive Energy (kJ mol^–1^) Per Molecule during 300 K NPT Simulation; Uncertainty Value Is the Standard Error of Fluctuations^a^

	cohesive energy (kJ mol^–1^)		
force field	starting in form I	starting in form IV	energy difference
expt.^65−69^	–87.65 to −98.58		
GAFF1	–81.3 ± 0.3	–82.6 ± 0.3	1.3 ± 0.6
GAFF2	–80.0 ± 0.4	–81.6 ± 0.4	1.6 ± 0.8
GAFF-D1	–113.5 ± 0.4	–118.2 ± 0.4	4.7 ± 0.8
GAFF-D3	–91.9 ± 0.4	–92.7 ± 0.4	0.8 ± 0.8
OPLS-AA	–87.5 ± 0.3	–87.5 ± 0.3	0.0 ± 0.7
OPLS-AA-N	–98.8 ± 0.3*	–117.5 ± 0.3**	18.7 ± 0.6
OPLS-AA-D	–83.7 ± 0.4	–83.7 ± 0.4	0.0 ± 0.8
OPLS-S	–79.3 ± 0.3	–79.4 ± 0.3	0.1 ± 0.6
OPLS-G	–80.2 ± 0.3	–80.2 ± 0.3	0.0 ± 0.6

a *Amorphous, ** New form.

Cohesive energies can be compared to experimental sublimation enthalpies at the same temperature. Experimental sublimation enthalpies of urea range from −87.65 to −98.58 kJ mol^–1^ at 298 K.^65−69^ GAFF-D3 is the only force field to obtain cohesive energies within the experimental range, which it does for both forms I and IV. OPLS-AA and OPLS-AA-D form I and IV simulations produce cohesive energies which are within 5% of the experimental values, as does the amorphous OPLS-AA-N simulation, although since this is amorphous, it is not appropriate to compare it to the crystal cohesive energy.

The cohesive energy of GAFF-D3 was previously calculated for different structures in vacuum including 8 × 8 × 8 and 20 × 5 × 5 supercells.^25^ It was found that the cohesive energy of the 8 × 8 × 8 supercell was within the experimental range, and the cohesive energy for the 20 × 5 × 5 supercell was close to the experimental values. This is in agreement with our work. In a comparison of CHARMM and the polarized SAPT-FF force fields the cohesive energies were found to be 101.2 and 99.0 kJ mol^–1^ for these, respectively.^18^

#### 3.1.3 Crystal Stability and the Effect of Temperature

Simulations of the bulk urea crystal were carried out at higher temperatures of 400, 450, and 500 K to investigate the effect of temperature on the crystal structure. We considered what happens to urea crystals that start in the form I structure, which is summarized in Table 7, and that start in the form IV structure, summarized in Table 8. The structure of both the amorphous solid and the melt consists of disordered molecules as is shown in Figure 3c, the difference between the two is that the molecules have translational and rotational mobility in the melt but not the amorphous solid, this was determined by visual inspection of the trajectory using VMD (Visual Molecular Dynamics^70^).

**Table 7:** Crystal Forms and Transitions of Bulk Crystal at Various Temperatures Starting from Form I

force field	300 K	400 K	450 K	500 K
GAFF1	form I	form IV	form IV	form IV
GAFF2	form I	form IV	form IV	form IV
GAFF-D1	form I	melt	melt	melt
GAFF-D3	form I	form I	form I	melt
OPLS-AA	form I	form I	form I	form I
OPLS-AA-N	amorphous	amorphous/Melt	melt	melt
OPLS-AA-D	distorted I/IV	distorted I/IV	distorted I/IV	melt
OPLS-S	distorted I/IV	distorted I/IV	distorted I/IV	melt
OPLS-G	distorted I/IV	distorted I/IV	distorted I/IV	melt

**Table 8:** Crystal Forms and Transitions of Bulk Crystal at Various Temperatures Starting from Form IV

force field	300 K	400 K	450 K	500 K
GAFF1	form IV	form IV	form IV	form IV
GAFF2	form IV	form IV	form IV	form IV
GAFF-D1	form IV	form IV	melt	melt
GAFF-D3	form IV	form I	form I	melt
OPLS-AA	form I	form I	form I	form I
OPLS-AA-N	new form	new form	new form	new form
OPLS-AA-D	distorted I/IV	distorted I/IV	distorted I/IV	melt
OPLS-S	distorted I/IV	melt	distorted I/IV	melt
OPLS-G	distorted I/IV	distorted I/IV	distorted I/IV	melt

**Figure 7:**
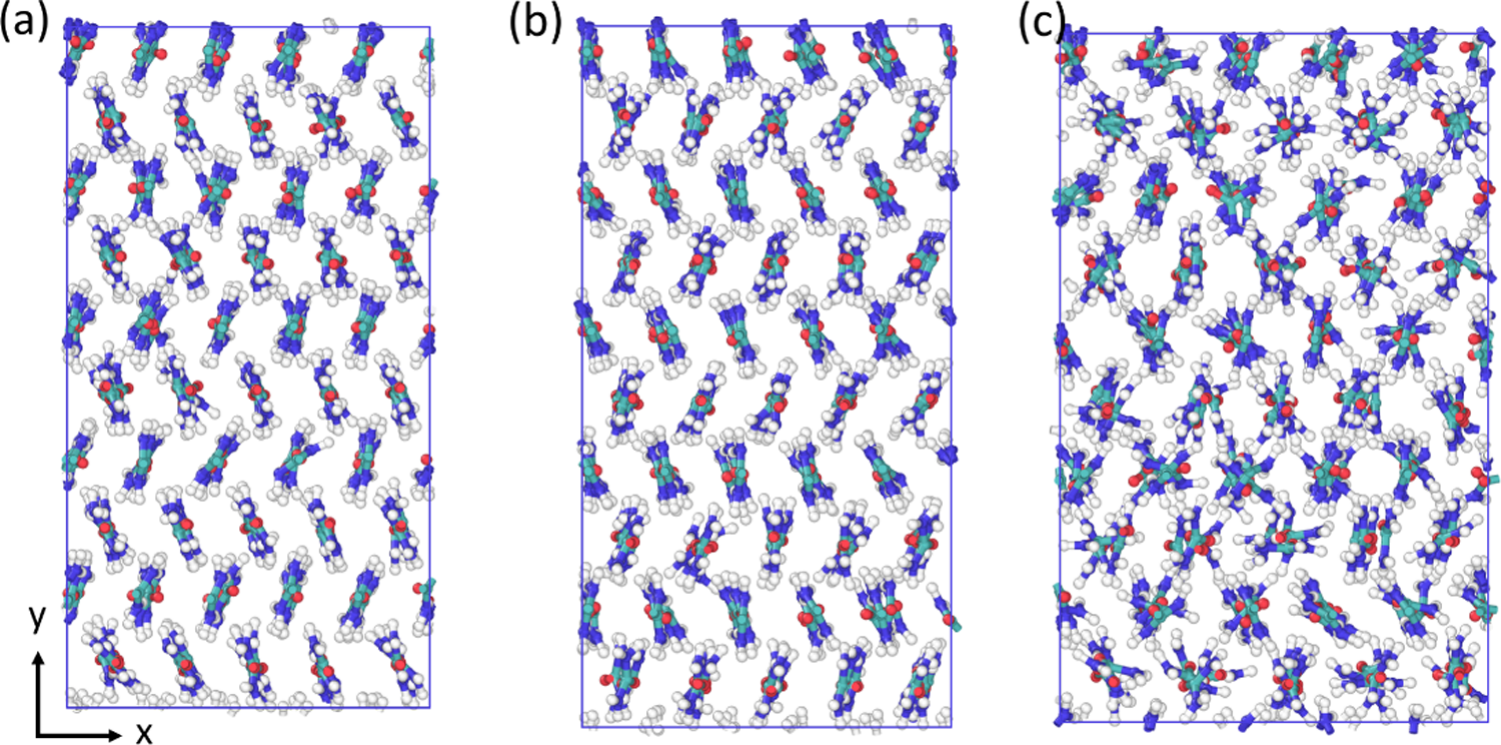
Snapshots of simulations run starting with the form IV crystal structure at higher temperatures. (a) “Left” herringbone alignment at 400 K for GAFF1 also representative of GAFF2, (b) “right” herringbone alignment at 400 K for GAFF1 also representative of GAFF2, and (c) some molecules spinning at 500 K for GAFF1 also representative of GAFF2.

There is some vibration of the molecules around the C=O axis (looking down the *z* direction) for all of the force fields, this can be seen in Figure 7a,b, the extent of this depends on the force field and also increases with temperature. This vibration is not the same for all of the molecules in the crystal; this results in the molecules in different layers not being perfectly aligned with the molecules above and below them.

Starting from form I, both the GAFF1 and GAFF2 crystals transformed to form IV at all higher temperatures. When starting from form IV, both GAFF1 and GAFF2 remain in the form IV structure, even at higher temperatures. The alignment of the form IV herringbone pattern inverts spontaneously during simulations at 400 K, changing between the structure in Figure 7a,b, this applies to GAFF1 and GAFF2 structures starting both from form I and IV. At higher temperatures (450 and 500 K), the molecules start to spin around their center, as illustrated in Figure 7c, for both structures starting from forms I and IV, which may indicate that this is close to melting.

Starting from form I, the GAFF-D1 crystal melted in the simulations at 400, 450, and 500 K. An additional simulation at 350 K was carried out, at which temperature GAFF-D1 did not melt and the molecules just vibrated around the C=O axis similarly to at 300 K. Starting from form IV the GAFF-D1 molecules showed some spinning at 400 K and the melted at 450 and 500 K. These much lower melting points compared to the GAFF1 and GAFF2 force fields indicate that this force field is stable for a smaller range of conditions.

When starting from form I, GAFF-D3 remained in form I but melted at 500 K. When starting from form IV, it transitioned to form I at 400 and 450 K, and melted at 500 K. It is interesting that GAFF-D3 seems to be more stable in form I, while the other GAFF force fields are not. There is a smaller difference between the cohesive energies of GAFF-D3 in the two structures than for the other GAFF force fields. This indicates that this behavior may be due to an entropic effect and that entropy dominates over enthalpy.

OPLS-AA reverts from form I to IV at all temperatures; for the higher temperature simulations, there are significant and increasing fluctuations of the crystal size around an average *a* = *b* value. Starting from form I the OPLS-AA-N system at 400 K is somewhere between the amorphous solid described above and a melt, and is a melt at 450 and 500 K. Starting from form IV the OPLS-AA-N system retains the new crystal form at all of the higher temperatures. For the OPLS-S, OPLS-G, and OPLS-AA-D crystals, all take on the distorted form I/IV structure, as described above, for 400 and 450 K starting from both form I and IV. The only exception to this is OPLS-S at 400 K starting from form IV which melts despite the simulation at 450 K not melting. This may indicate that some small instability led to melting which has not been observed in any of the other simulations due to short simulation times. All of the OPLS-S, OPLS-G, and OPLS-AA-D crystals melt in the simulations at 500 K.

We note that the experimental melting point of urea is 406 K. We observe that most of the force fields did not result in melting of the bulk urea crystal at 400 or 450 K, with some not melting even at 500 K. However, periodic boundaries make the crystal an infinite lattice with no edges, and therefore there is a superheating phenomenon leading to significant overestimation of the melting point.^71^ For this reason, melting points are not accurately determined by simply heating a bulk crystal. The purpose of these simulations at increased temperatures is to gain insight into the relative stabilities of the force fields compared. However, other methods such as studying the crystal-melt interface can be used to more accurately gauge the melting point of a system, this has been used for GAFF1 urea where the melting point was found to lie between 400 and 420 K.^11^ Crystallites can also be used to test melting properties instead of the bulk crystal; this is likely to lead to an underestimation of the melting point. This has been carried out with GAFF-D3 for an 8 × 8 × 8 unit cell cubic crystal, by Özpınar et al.^25^ who found that crystal melted completely at 385 K.

From the above results of the behavior at increased temperatures, it appears that GAFF-D3 and OPLS-AA are the only force fields that are stable in form I at most temperatures. OPLS-AA is the most consistent force field with the same structure and cohesive energy obtained regardless of the starting structure. It also retains the same structure at all of the temperatures; however, there are significant fluctuations around the average lattice lengths. At 300 K GAFF-D3 is more stable in form IV based on the cohesive energy; however, it is only in simulations at 400 K and above that a spontaneous change from form I to IV is observed. OPLS-G, OPLS-S, and OPLS-AA-D are stable in a distorted form I/IV structure. GAFF1 and GAFF2 crystals are more stable in form IV than form I, this is consistent with the cohesive energies obtained and the findings of Francia et al.^63^ GAFF-D1 melts at higher temperatures, and OPLS-AA-N also exhibits melting or a new crystal form, not observed for urea.

Based on all of the results for the crystal structures, we conclude that GAFF-D3 and OPLS-AA are the most suitable force fields for modeling the form I urea crystal. The performance of form I density and lattice parameters is very similar between these two force fields; however, GAFF-D3 experiences smaller fluctuations in the size of the supercell. The cohesive energy of GAFF-D3 is closer to the experimental values than that of OPLS-AA, but GAFF-D3 has a lower cohesive energy for form IV instead of the expected form I. GAFF-D3 can be used to model urea in the high-pressure form IV form at ambient conditions but has a preference for the form I structure at increased temperatures, whereas OPLS-AA converts from form IV to I even at ambient conditions.

### 3.2 Solution Properties

In this section, we investigate the properties of urea aqueous solutions over a large concentration range, compared to experiments and simulations from the literature, which are limited to undersaturated concentrations. Our simulations extend into the supersaturated concentration region, as this is relevant to studies of urea crystallization. The highest concentration studied was 1000 urea molecules in 1000 water molecules, which is 3334 g kg^–1^, which corresponds to a supersaturation of 2.78 at 300 K, based on experimental solubility.^61^ We present the solution density, radial distribution functions, and diffusion properties.

#### 3.2.1 Solution Density

**Figure 8:**
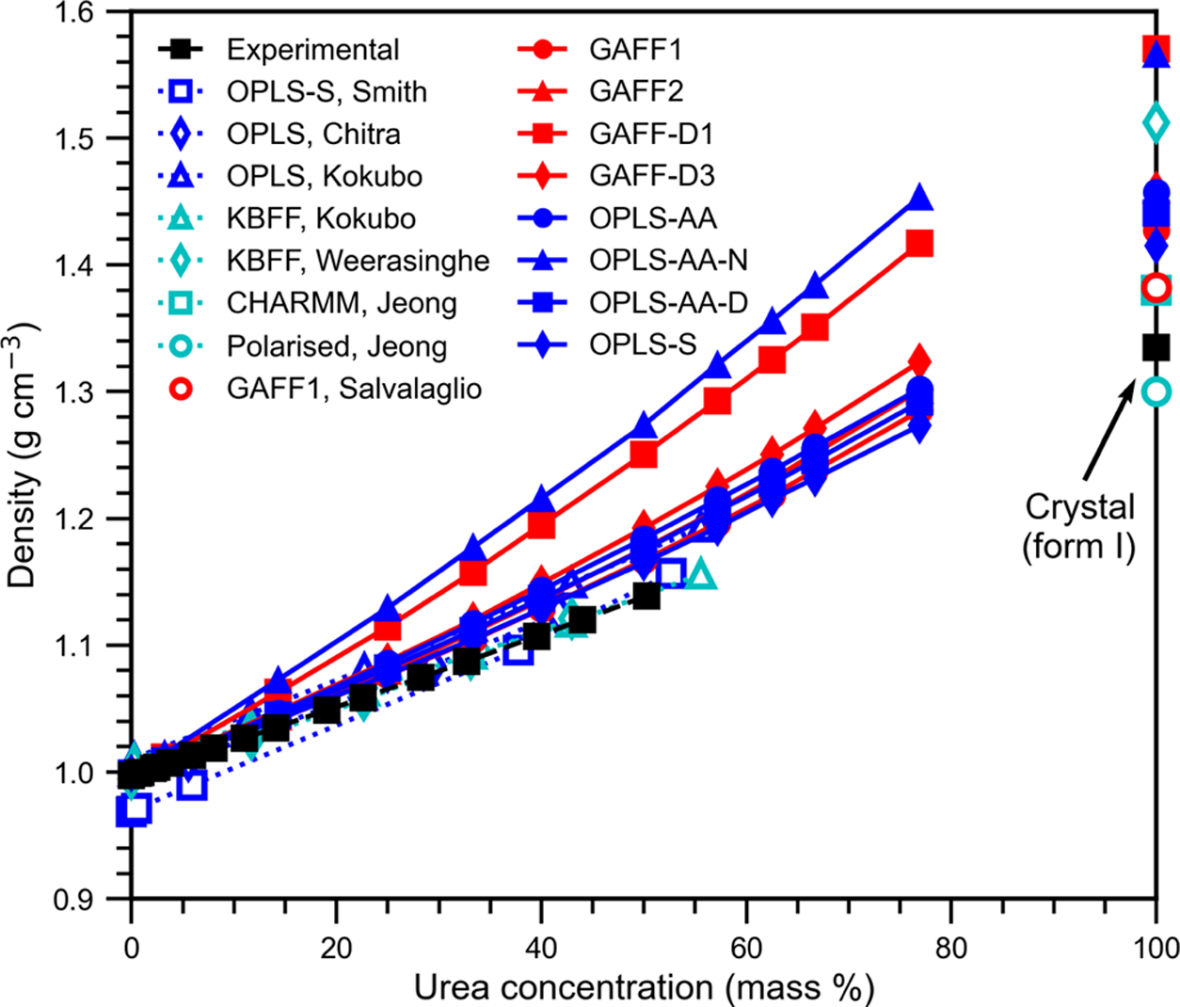
Urea solution density. OPLS-S overlaps OPLS-G (not shown). Error bars, representing the standard deviation, are smaller than the symbols. Tabulated values with the standard deviation are given in the Supporting Information. The key gives the urea force field and the literature source where appropriate. The literature references are experimental solution density (Gucker et al.^72^) and experimental crystal form I density (Mullin,^61^) and simulated solution and crystal results (Smith et al.,^23^ Chitra and Smith,^20^ Kokubo and Pettitt,^16^ Weerasinghe and Smith,^26^ Jeong et al.^18^ and Salvalaglio et al.^11^).

Time-averaged solution densities are shown in Figure 8 and compared to experimental data and simulation results from the literature. Tabulated values of our results with the standard deviation are given in the Supporting Information. For the pure urea density, we used crystal form I densities at 300 K. The solution densities obtained are close to the experimental values at low concentrations but deviate increasingly from experimental values at higher concentrations. This is not surprising since the SPC/E water force field used here reproduces well the experimental density of water, which gives a density of 0.9993 ± 0.0008 g cm^–3^ at 300 K, compared to the experimental density of 0.997 g cm^–3^. However, the urea crystal density is overestimated by all of the force fields, leading to deviations at high urea concentrations.

For the solution phase, OPLS-S, OPLS-G, and GAFF1 perform the best. Note that it is not possible to distinguish the OPLS-S and OPLS-G curves from each other since these force fields are very similar. The most concentrated urea solution for which experimental data is available is 50 mass %, at this concentration, all force fields except GAFF-D1 and OPLS-AA-N perform well, overestimating the density by less than 5%. GAFF1, OPLS-S, and OPLS-G perform very well overestimating the concentration by less than 3%, GAFF2 and OPLS-AA-D overestimate by less than 4%, and GAFF-D3 and OPLS-AA overestimate by less than 5%. Only GAFF-D1 and OPLS-AA-N significantly overestimate the density by almost 10 and 12%, respectively.

Data from Smith et al.^23^ is based on the OPLS-S force field coupled with the SPC water model, which underestimates the density of pure water at 300 K. Thus, the SPC water model causes the density of the low-concentration solutions to be underestimated, while for higher concentrations, their OPLS-S simulations reproduce the experimental density values more closely than our work. However, the gradient of the density with concentration in our work is lower than that of Smith et al.^23^ and more similar to the experimental gradient. Chitra and Smith^20^ use a combination of the nonbonded OPLS urea parameters with the bonded CHARMM parameters, with the SPC/E, which leads to a very close reproduction of the experimental density. Kokubo and Pettitt^16^ use only the nonbonded OPLS urea parameters which lead to density values similar to those obtained in this work, whereas their use of KBFF leads to the most accurate density reproduction.

This shows that the combination of water and urea (solvent and solute) force fields influences the solution density, as expected. Apart from GAFF-D1 and OPLS-AA-N, all of the other GAFF and OPLS force fields tested here, as well as the additional ones from Chitra and Smith^20^ and Kokubo and Pettitt,^16^ reproduce well the density of aqueous urea solutions.

#### 3.2.2 Radial Distribution Functions

Now we turn to the solution structure. Two different atomic radial distribution functions (RDFs) are discussed here: O–H_W_, and O–N, where the subscript ‘W’ indicates that the hydrogen belongs to a water molecule and atoms without subscripts belong to urea. Additional RDFs for the H–O_W_, O–O_W_, N–H_W_, N–O_W_, and C–O_W_ interactions are presented in the Supporting Information. These are compared to RDF curves from the literature, obtained from both experiment and simulation. Two RDFs are shown for each atom pair, a dilute one (3.23%mass) and a more concentrated one (50.0%mass). The dilute reference RDFs are from Duffy et al.^15^ at 1.24% mass and Ishida et al.^73^ where the concentration was just referred to as “dilute”. The concentrated reference RDFs are at 43.0%mass, 45.5%mass, and 58.8%mass, for Weerasinghe and Smith,^26^ Soper et al.^46^ and Burton et al.,^74^ respectively. Two of the literature RDFs were obtained experimentally, Burton et al.^74^ used neutron scattering and Soper et al.^46^ used neutron diffraction with empirical potential structure refinement with the OPLS-Urea urea and SPC/E water models to process the results. The other RDFs were obtained from molecular dynamics simulations with the following force fields: Duffy et al.^15^ used OPLS-Urea with the TIP4P water model; Ishida et al.^73^ used the RISM-SCF method (reference interaction site model–self-consistent-field) for urea with the SPC water model; and Weerasinghe and Smith^26^ used the KBFF urea model with the SPC/E water model.

**Figure 9:**
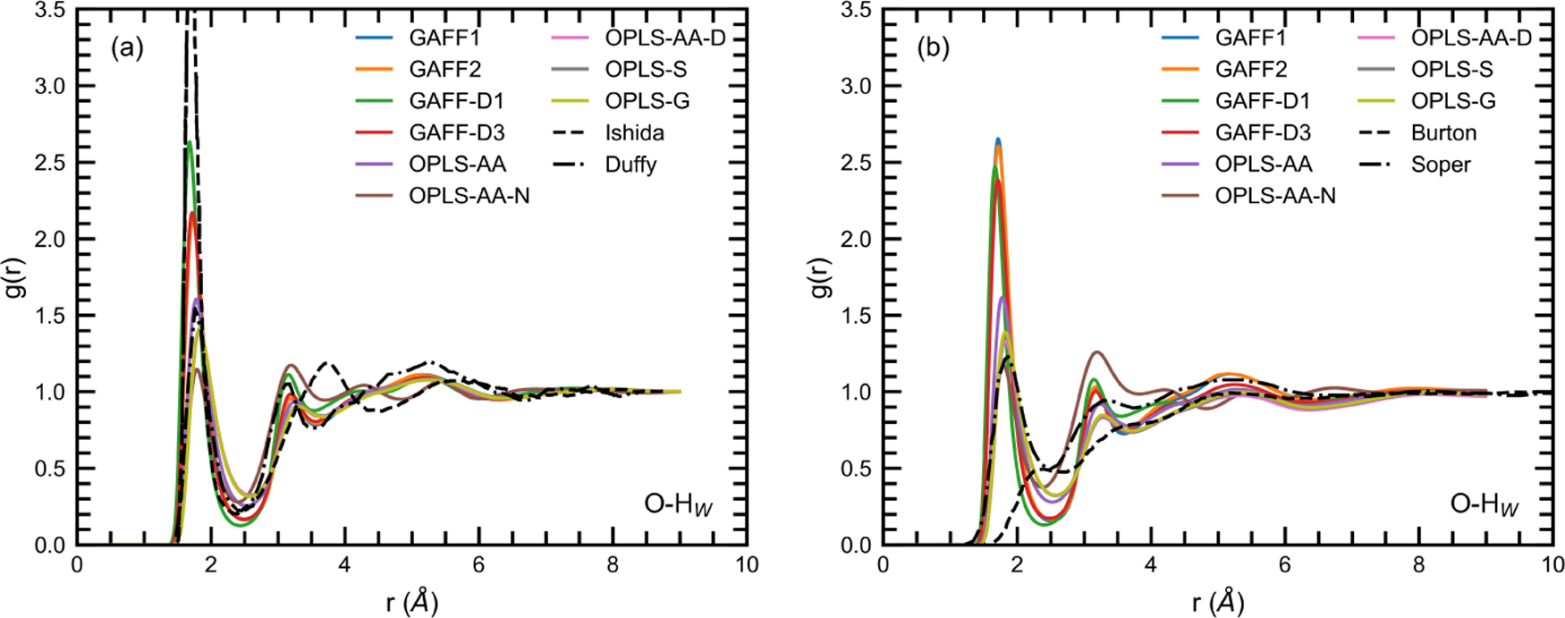
O–H_W_ RDFs for (a) the dilute solutions and (b) the concentrated solutions. Literature data is taken from Ishida et al.,^73^ Duffy et al.,^15^ Burton et al.,^74^ and Soper et al.^46^

The O–H_W_ RDFs are shown in Figure 9, and the shapes are similar for both the dilute and concentrated solutions. There is a first peak just below 2 Å, indicating that strong hydrogen bonding between urea and water is present. There are also weaker second and third peaks appearing at around 3 and 5 Å, respectively. The O–H_W_ RDFs are similar to those obtained by Duffy et al.,^15^ but differ from Ishida et al., which have a second peak at around 3.5 Å. For the concentrated solution, the main difference between the different force fields is that the first peak is significantly higher for the four GAFF force fields. The OPLS RDFs are similar to Soper et al.^46^ but differ from Burton et al.,^74^ which have a weak first peak at 2.5 Å and a barely noticeable second peak.

**Figure 10:**
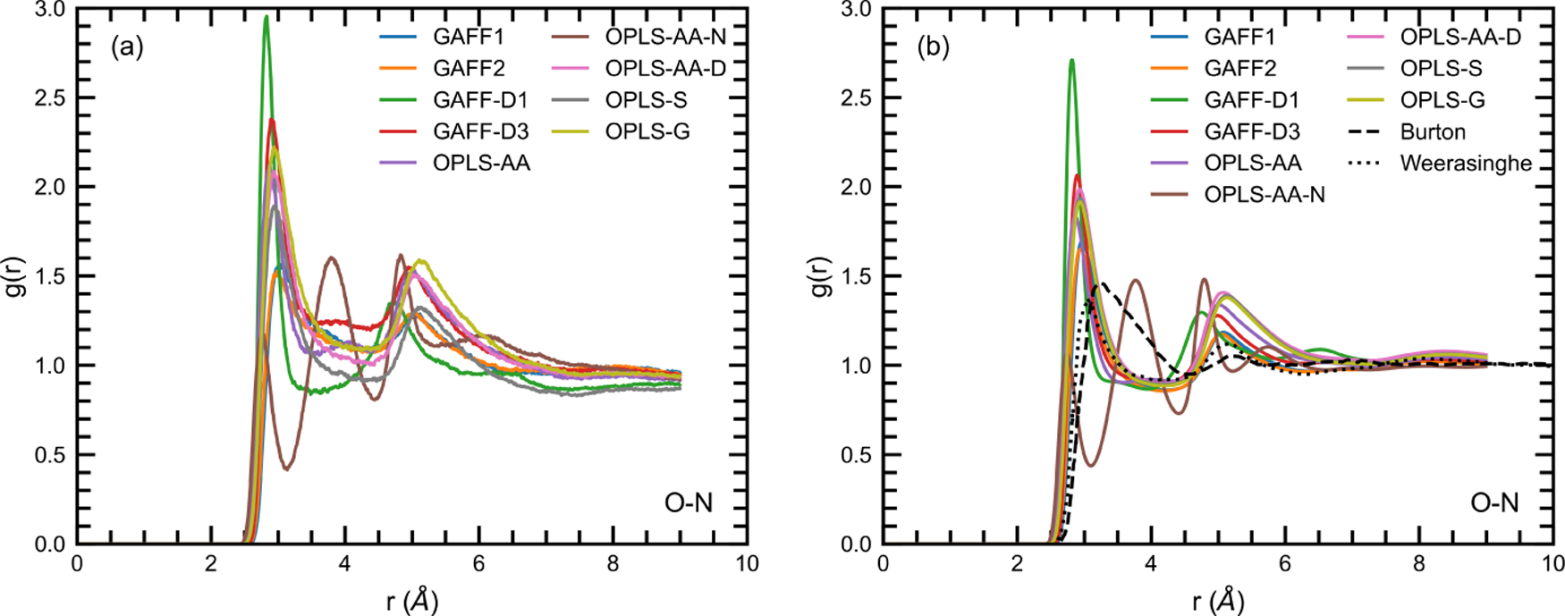
O–N RDFs for (a) dilute solutions and (b) concentrated solutions. Literature data is taken from Burton et al.^74^ and Weerasinghe and Smith.^26^

The O–N RDFs, shown in Figure 10, provide insight into the urea–urea interactions both within the same molecule and between different molecules. The RDFs have two peaks at around 3 and 5 Å. The location of the first peak corresponds to the interaction between O and N molecules within the same molecule, so this should be similar for all of the solutions regardless of concentration. Our RDFs of these materials are similar to those of Duffy et al. and Weerasinghe and Smith. The first peak for Burton et al. fell much more slowly in the concentrated solution. Unusually the first peak is much lower for OPLS-AA-N compared with the rest of the force fields. However, there is an extra peak between the first and second for OPLS-AA-N at >3.5 Å and the second peak is shifted forward to >4.5 Å. This may be related to the partial charge difference between the O and N atoms, which is significantly greater for OPLS-AA-N compared to all of the other force fields.

Overall our results compare well with literature sources, with slight variation between the different force fields, with the exceptions of GAFF-D1 and particularly OPLS-AA-N which do not reproduce the urea solution structure well. In general, the partial charges and the charge differences between two atoms have a small, but noticeable, effect on the RDF structure. GAFF1 and GAFF2 have a shared charge set, as do OPLS-G, OPLS-S, and OPLS-AA-D, and where the height and position of the peaks vary slightly between the different force fields, the peaks are generally very similar within each of these two groups. In general, when comparing the first peak of each RDF for the different force fields, the peaks at the lowest *r* values are taller and narrower than the corresponding peaks at slightly larger *r* values.

#### 3.2.3 Diffusion Coefficients

**Figure 11:**
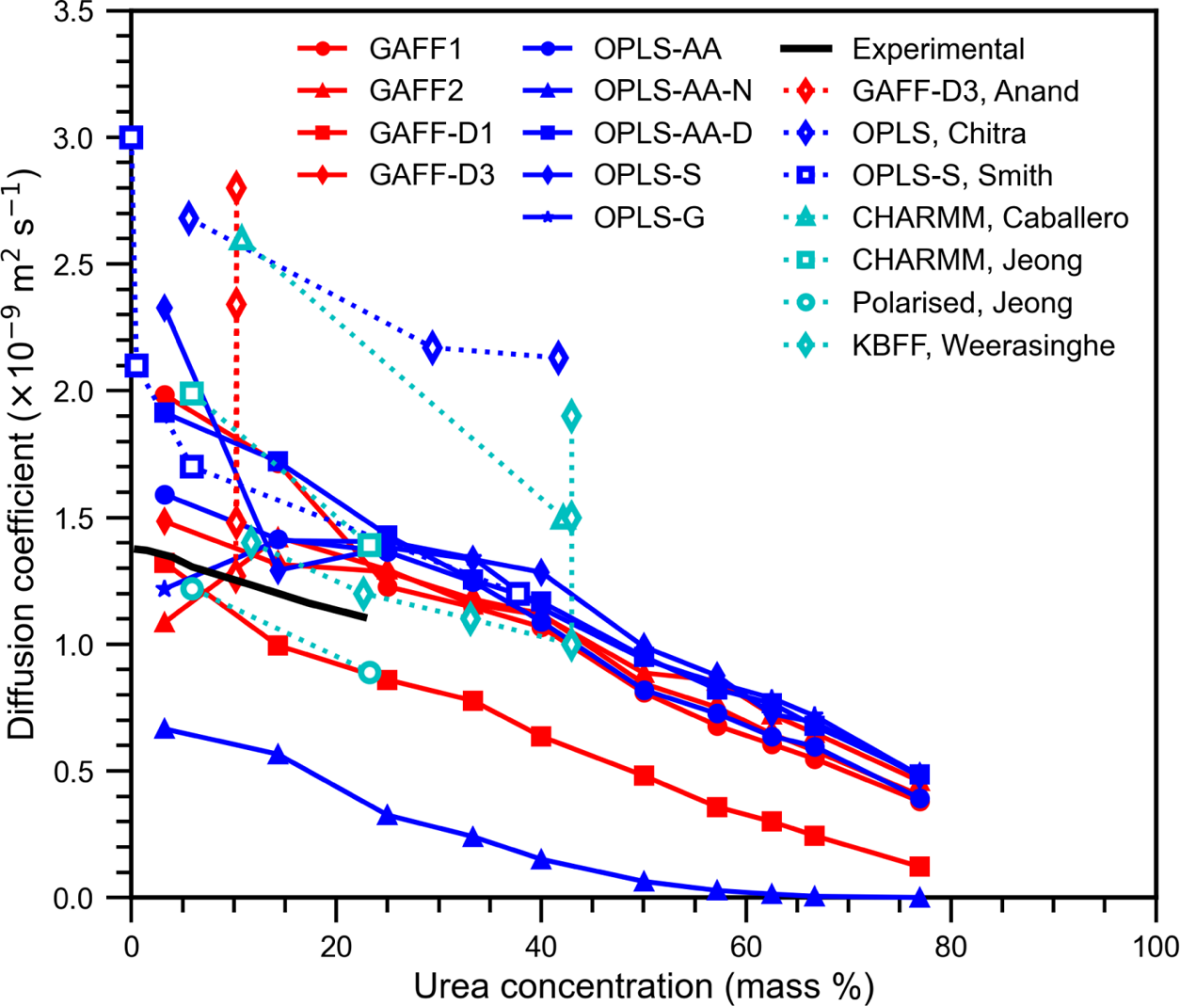
Diffusion coefficients for urea in aqueous solution over a range of concentrations. Error bars, representing the standard error are smaller than the symbols. Tabulated values with the standard error are given in the Supporting Information. The key gives urea force field and the literature source where appropriate. The literature references are experimental by Albright and Mills,^75^ calculated by Anand and Patey^14^ with TIP3P, SPC, SPC/E, and TIP4P water from top to bottom, Chitra and Smith^20^ with SPC/E water, Smith et al.^23^ with SPC water, Caballero-Herrera and Nilsson^17^ with TIP3P water, Jeong et al.^18^ with TIP3P water for CHARMM and SWM4-NDP water with SAPT-FF (polarized), and Weerasinghe and Smith^26^ with SPC/E water and also TIP3P and SPC water (top and middle) at their highest concentration.

Finally, we compare how the various force fields describe the solution dynamics. Calculated diffusion coefficients for urea in aqueous solution, at 300 K, are shown in Figure 11 and compared to experimental data and simulation results from the literature. Tabulated values of our results with the standard deviation are given in the Supporting Information.

The diffusion coefficients decrease with increasing concentration as the solution becomes more densely packed with urea molecules. Generally, the GAFF force fields more closely reproduce the experimental urea diffusion coefficient than the OPLS force fields do. GAFF-D3 most closely matches the experimental diffusion coefficients followed by GAFF-D1 and OPLS-AA. Most force fields studied here overestimate the diffusion coefficient; however, GAFF-D1 and OPLS-AA-N underestimate the diffusion coefficient. These two force fields predicted the highest solution densities, meaning that the solution is more closely packed and reduces the molecular diffusion in the solution.

Our results are within the range of the literature results from other simulations. The results from Anand and Patey^14^ used the GAFF-D3 urea force field and four different water force fields (TIP3P, SPC, SPC/E, and TIP4*P*/2005) and show how significantly the choice of water force field can affect the diffusion coefficient of a solution. Their GAFF-D3 and SPC/E force field combination matched very well with our values obtained for the same force field combination at similar concentrations. Similarly, the results from Smith et al.^23^ for OPLS-S and SPC are very close to our results from OPLS-S and SPC/E. The large variation in the performance of diffusion coefficients from the literature can arise from simulation size, exact calculation method, and region of MSD data used to extract the diffusion coefficient. For more accurate estimation of diffusion coefficient, a series of simulations at different system sizes should be used.^76^

Overall, looking at the solution results, all of the force fields perform relatively well, with the exception of GAFF-D1 and OPLS-AA-N. The OPLS force fields perform slightly better for solution density, the GAFF force fields perform slightly better for urea diffusion coefficients, and there is no clear distinction when looking at the RDFs. The GAFF-D1’s poor performance follows from its high crystal density, and OPLS-AA-N can reproduce neither realistic crystal nor solution structures and behaviors. For OPLS-AA-N this is disappointing since it is much more user-friendly to use the LigParGen software to obtain the force field parameters than to manually go through the lists of parameters for OPLS-AA which are published across several articles. We note that this may be specific for urea, possibly due to its small size, since other smaller organic molecules have successfully been parametrized using LigParGen.^52^

## 4 Conclusions

In this work, we have compared the bulk crystal and solution properties of urea for four GAFF force fields and five OPLS force fields. Parametrization of partial charges was done using the Antechamber software^32^ for GAFF1 and GAFF2, and using LigParGen^43−45^ for OPLS-AA-N and manually taking published parameters from Özpınar et al.^24^ for GAFF-D1 and GAFF-D3, from Jorgensen et al.^4^ and Weiner et al.^5^ for OPLS-AA, from Smith et al.^23^ for OPLS-S, also consulting Oostenbrink et al.^8^ for OPLS-G and Duffy et al.^15^ for OPLS-AA-D. The SPC/E model was selected for water since other studies have already concluded that it^14,50,52^ is good for modeling solution properties including density and diffusion and it can successfully be paired with a range of other force fields including both GAFF and OPLS.^14,50,52^

The bulk crystal simulations were carried out at 300, 400, 450, and 500 K for each force field, starting from both forms I and IV, with one additional simulation at 350 K for GAFF-D1 form I, leading to 73 bulk crystal simulations. Starting from crystal form I, at 300 K all four GAFF force fields and OPLS-AA retain the form I structure. The OPLS-AA-N crystal collapses into an amorphous solid, while the remaining OPLS force fields form a distorted form I/IV crystal structure, which has previously been observed with OPLS-S^9^ (this structure also has similarities to the form IV urea crystal structure). Starting from crystal form IV, which is obtainable only experimentally at high pressures, the GAFF force fields retain this crystal structure. OPLS-AA transforms to form I, OPLS-AA-N transforms into a new crystal form, while the remaining OPLS force fields take on the same distorted crystal form that was observed when starting from form I.

At 300 K all of the force fields overestimate the experimental form I crystal density by 7–19%; in contrast, the high-pressure form IV density is underestimated by 4–17%. The form I density is best reproduced by OPLS-S and OPLS-G in the distorted form I/IV structure with the worst performance from GAFF-D1 and OPLS-AA-N. The cohesive energies of forms I and IV are very close to each other, with form IV being marginally more stable at 300 K. The crystal cohesive energy, compared to the experimental sublimation, was accurately reproduced by GAFF-D3 with OPLS-AA and OPLS-AA-D also performing well. The stability of the bulk crystals was tested at higher temperatures of 400, 450, and 500 K, although these simulations were not intended to give accurate estimates of the melting point. This found that GAFF1 and GAFF2 have a preference for the form IV structure (with form I to IV transitions); contrastingly, GAFF-D3 and OPLS-AA prefer form I (with form IV to I transitions), OPLS-AA-D, OPLS-S and OPLS-G retain the distorted form I/IV crystal structure, and GAFF-D1 melts since it is not stable at higher temperatures. These tests on the bulk crystal indicate that GAFF-D3 and OPLS-AA are the most suitable force fields for modeling urea crystals. GAFF-D3 accurately reproduces the crystal form at 300 K and the cohesive energy as well as favoring form I compared to form IV at higher temperatures. OPLS-AA performs very similarly to GAFF-D3, it has a slightly lower cohesive energy, and it is most stable in form I for all simulation conditions.

Ten different solution concentrations were studied for each force field, with one additional simulation of pure water, leading to 91 simulations. The range of concentration was varied from very dilute (33.34 g kg^–1^) to highly supersaturated solutions (3334 g kg^–1^). All of the force fields reproduced the aqueous solution density well, apart from GAFF-D1 and OPLS-AA-N, which significantly overestimated the density even at low urea concentrations. Radial distribution functions showed that all of the force fields, with the exception of OPLS-AA-N, and to some extent GAFF-D1 give the structure of urea solutions in good agreement with the literature. The diffusion coefficients of the solution were reproduced reasonably well by all of the force fields, again with the exception of OPLS-AA-N, which significantly underestimated the diffusion coefficient, indicating that there was very little to no diffusion taking place in any of the supersaturated solutions. Based on the properties we have studied, GAFF1, GAFF2, GAFF-D3, OPLS-AA, OPLS-AA-D, OPLS-S, and OPLS-G all perform similarly.

OPLS-AA-N is essentially a newer version of the OPLS-AA force field based on the same bonded and Lennard-Jones parameters but with a few small changes. However, where OPLS-AA has predetermined partial charges for each atom type, OPLS-AA-N instead calculates these charges based on the molecular structure. For the case of urea, the partial charges and charge dipoles within the molecule differ significantly between the two force fields. The partial charges make OPLS-AA-N unsuitable for modeling urea, either in the crystal or solution state.

We conclude that the best overall performing force fields are GAFF-D3 and OPLS-AA, which have good properties in both the crystal and the solution phases. GAFF-D3 accurately reproduces the crystal cohesive energy and high-temperature behavior of the crystal including predicting the stability of the form I crystal structure; this is also the best-performing force field based on the diffusion coefficients calculated. OPLS-AA has good overall crystal properties with a preference for the stable form I crystal structure in all conditions tested and is the best-performing force field based on the solution density. The better performance of GAFF-D3 compared to the other GAFF force fields shows that for GAFF, a molecule-specific charge optimization is worthwhile, which has also been noted for other druglike organic molecules.^50,52^ Conversely, for the OPLS force fields, the standard OPLS-AA force field performed well, and adding the urea-specific charges of OPLS-AA-D or OPLS-AA-N was not advantageous. This highlights the sensitivity of systems to small changes in force fields and the importance of validating the force field for intended applications before use.

More generally, we have discussed the importance of performing force field validation tests at the outset of new studies. For the application to crystallization processes, both crystal and solution properties should be tested. We have suggested the use of simple bulk crystal simulations to test the crystal structure, density, and cohesive energy as well as bulk solution simulations to test solution density and diffusion coefficients. All of these properties can easily be obtained from short test simulations and compared to experimental data. We also note that clearly reporting the force field and simulation parameters used is important to enable the reproducibility of published work. By continuing to develop the use of MD to model real systems and improve the force fields available for use, we can begin to use MD to carry out more complex studies, including predictive experiments in areas such as drug design, which will enable better overall use of resources in the research and development field.

## Supporting Information Available

Full force field details of equations and parameters; tabulated values of results and properties only presented graphically; and further radial distribution functions plots with analysis. Note, simulation input files are available from https://doi.org/10.15129/580aa404-0d2d-448d-b728-4b33e33e0c1b (PDF)
