# Supplementary material for: Assessment of GAFF and OPLS Force Fields for Urea: Crystal and Aqueous Solution Properties
Source: Cryst Growth Des. 2023 Dec 8;24(1):143–58. doi: 10.1021/acs.cgd.3c00785 (PMC10767702; doi:10.1021/acs.cgd.3c00785)
Supplement: Supplementary file 1 — cg3c00785_si_001.pdf [file cg3c00785_si_001.pdf]

Supporting Information for Assessment of GAFF and OPLS force fields for urea: crystal and aqueous solution properties

Samira Anker,[†,‡] David McKechnie,[†,‡] Paul Mulheran,[†] Jan Sefcik,[†,‡] and Karen Johnston[*,†]

†*Department of Chemical and Process Engineering, University of Strathclyde, Glasgow, UK*

‡*Future Continuous Manufacturing and Advanced Crystallisation Research Hub, University of Strathclyde, Glasgow, UK*

E-mail: karen.johnston@strath.ac.uk

A Force field parameters and equations

This section provides all the force field equations for bonded and non-bonded interactions used by all the force fields studied. This is followed by tables with the parameters that have been used for each of the force fields.

A.1 Force field equations

The same force field equations are used for all of the GAFF force fields (GAFF1, GAFF2, GAFF-D1 and GAFF-D3. The same non-bonded equations are used for all of the OPLS force fields but the bonded equations differ. OPLS-AA, OPLS-AA-N and OPLS-AA-D use the bonded AMBER equations, where as OPLS-S and OPLS-G use the bonded GROMOS equations.

A.1.1 Bond interactions

GAFF, OPLS-AA, OPLS-AA-N and OPLS-AA-D use a harmonic bond potential, given in Eq. 1, which is implemented with *bond_style harmonic* in LAMMPS. This is also used by the SPC/E water model. OPLS-S and OPLS-G use the gromos bond potential, given in Eq. 2, which is implemented with *bond_style gromos* in LAMMPS.

$$E_{\text{bond}} = K_r \, (r - r_0)^2 \tag{1}$$

$$E_{\text{bond}} = K_r \, (r^2 - r_0^2)^2 \tag{2}$$

K_r is the bond constant, r_0 is the equilibrium distance and r is the distance between the two atoms.

A.1.2 Angle interactions

GAFF, OPLS-AA, OPLS-AA-N and OPLS-AA-D use a harmonic angle potential, given in Eq. 3, which is implemented with *angle_style harmonic* in LAMMPS. This is also used by the SPC/E water model. OPLS-S and OPLS-G use a squared cosine angle potential, given in Eq. 4, which is implemented with *angle_style cosine/squared* in LAMMPS.

$$E_{\text{angle}} = K_\theta \, (\theta - \theta_0)^2 \tag{3}$$

$$E_{\text{angle}} = K_\theta \, (\cos(\theta) - \cos(\theta_0))^2 \tag{4}$$

K_θ is the angle constant, θ_0 is the equilibrium angle and θ is the angle between the three atoms.

A.1.3 Dihedral interactions

GAFF uses a fourier dihedral potential, given in Eq. 5, which is implemented with *dihedral_style fourier* in LAMMPS. OPLS-AA, OPLS-AA-N and OPLS-AA-D use the opls di-

hedral potential, given in Eq. 6, which is implemented with *dihedral_ style opls* in LAMMPS. OPLS-S and OPLS-G use a harmonic dihedral potential, given in Eq. 7, which is implemented with *dihedral_ style harmonic* in LAMMPS.

$$E_{\text{dihedral}} = K_{\phi,i}\ [1.0 + \cos(n_i\phi\ - \delta_i)] \tag{5}$$

$$E_{\text{dihedral}} = \frac{1}{2}V_1\ [1.0{+}\cos(\phi)] + \frac{1}{2}V_2\ [1.0{-}\cos(2\phi)] + \frac{1}{2}V_3\ [1.0{+}\cos(3\phi)] + \frac{1}{2}V_4\ [1.0{-}\cos(4\phi)] \tag{6}$$

$$E_{\text{dihedral}} = K_{\phi}\ [1.0 + d\ \cos(n\phi)] \tag{7}$$

$K_{\phi,i}$ and V_{1-4} are the force constants, ϕ is the dihedral angle, n is the periodicity of torsion, δ is the phase angle and $d = \cos(\delta)$. These three equations are describing the same potential in different ways: 7 is simply a re-arranged version of Eq. 5 and Eq. 6 is a re-arranged and expanded version of Eq. 5.

A.1.4 Improper dihedral interactions

GAFF and OPLS-AA, OPLS-AA-N and OPLS-AA-D use a fourier improper potential, given in Eq. 8, which is implemented with *improper_ style cvff* in LAMMPS. This is the potential that was used for the GAFF dihedrals. OPLS-S and OPLS-G uses a harmonic improper potential, given in Eq. 9, which is implemented with *improper_ style harmonic* in LAMMPS.

$$E_{\text{improper}} = K_{\xi}\ [1.0 + d\ \cos(n\xi)] \tag{8}$$

$$E_{\text{improper}} = K_{\xi}\ (\xi - \xi_0)^2 \tag{9}$$

K_{ξ} is the force constant, ξ is the improper angle, n is the periodicity of torsion and $d = \cos(\delta)$ where δ is the phase angle.

A.1.5 Non-bonded interactions

All the force fields use the Coulomb potential for the non-bonded electrostatic interactions, this is given in Eq. 10.

$$E_{\text{Coul}} = \sum \frac{q_i q_j}{4\pi\epsilon_0 r} \tag{10}$$

q is the charge, i and j are the two different atoms, r is the distance between atoms i and j and ϵ_0 is the dielectric constant with a default value of 1.0 for a vacuum.

All the force fields use the Lennard-Jones potentials for the non-bonded dispersion interactions, this is given in Eq. 11.

$$E_{\text{LJ}} = 4\varepsilon_{ij} \left[\left(\frac{\sigma_{ij}}{r}\right)^{12} - \left(\frac{\sigma_{ij}}{r}\right)^{6} \right] \tag{11}$$

Again, i and j are the two atoms interacting and r is the distance between these atoms. ε is the minimum energy between the two atoms and σ is the distance required between the two atoms for the energy to be zero.

The ε and σ values are specified for the interactions between atoms of the same type (e.g. C - C interactions). Mixing rules are used to calculate these values for any atom pairing (e.g. C - O and C - N). For this, all the GAFF force fields use the arithmetic (Lorentz Berthelot) mixing rules given in Eqs. 12 and 14 and all the OPLS force fields use the geometric mixing rules given in Eqs. 13 and 14. For water, the mixing rules were chosen to match the other force field being used.

$$\sigma_{ij} = \frac{1}{2}(\sigma_i + \sigma_j) \tag{12}$$

$$\sigma_{ij} = (\sigma_i \sigma_j)^{\frac{1}{2}} \tag{13}$$

$$\varepsilon_{ij} = (\varepsilon_i \varepsilon_j)^{\frac{1}{2}} \tag{14}$$

A.2 GAFF parameters for urea

The GAFF1 and GAFF2 parameters are taken from GAFF version 1.81 and 2.11 respectively, these were obtained using the Antechamber software which is part of AmberTools21.[1] The GAFF-D1 and GAFF-D3 parameters were taken from Özpınar et al.[2].

Table 1: GAFF parameters for mass in Da and charge in e.

Atom type	Mass	Charge		
	GAFF[1,3]	GAFF1,[1,3] GAFF2[1]	GAFF-D1[2]	GAFF-D3[2]
C	12.01	0.7261	1.172	0.884
O	16.00	-0.6391	-0.795	-0.660
N	14.01	-0.6420	-1.098	-0.888
H	1.008	0.2990	0.454	0.388

Table 2: The GAFF Lennard-Jones parameters used for urea. ε in kcal mol^{-1} and σ in Å. The σ (Lennard-Jones diameter) values were given as R_{min} (van der Waals radius) values, $\sigma = 2^{\frac{5}{6}} R_{\mathrm{min}}$ was used for the conversion (from $2R_{\mathrm{min}} = 2^{\frac{1}{6}}\sigma$).

Atom type	GAFF1,[1,3] GAFF-D1,[2] GAFF-D3[2]		GAFF2[1]	
	ε	σ	ε	σ
C	0.0860	3.3997	0.0988	3.3152
O	0.2100	2.9599	0.1463	3.0481
N	0.1700	3.2500	0.1636	3.1809
H	0.0157	1.0691	0.0100	1.1065

A.3 OPLS parameters for urea

Table 3: The GAFF bond and angle parameters for urea. K_r in kcal mol^{-1} Å^{-2}, r_0 in Å, K_θ in kcal mol^{-1} rad^{-2} and θ_0 in °.

	GAFF1[1,3]		GAFF2[1]		GAFF-D1, GAFF-D3[2]	
Bonds	K_r	r_0	K_r	r_0	K_r	r_0
C - O	637.7	1.2183	652.57	1.218	656	1.250
C - N	427.6	1.3789	356.21	1.379	424	1.383
N - H	403.2	1.0129	527.31	1.013	434	1.010
Angles	K_θ	θ_0	K_θ	θ_0	K_θ	θ_0
C - N - H	48.33	117.55	48.691	117.550	30	120.0
N - C - O	74.22	123.05	113.811	123.050	80	120.9
N - C - N	72.90	113.56	112.428	113.560	70	118.6
H - N - H	39.56	117.95	39.011	117.950	35	120.0

Table 4: The GAFF dihedral and improper dihedral parameters. K_ϕ in kcal mol^{-1}, n is unit less and d in °. These parameters are the same for all the force fields[1–3] with one exception. *The H-N-C-N dihedral is not used in GAFF-D1 and GAFF-D3.[2,3]

Dihedrals	K_ϕ	n	d
H - N - C - O	2.50	2	180.0
H - N - C - O	2.00	1	0.0
H - N - C - N*	2.50	2	180.0
Improper dihedrals	K_ϕ	n	d
C - N - H - H	1.1	2	180.0
O - C - N - N	10.5	2	180.0

Table 5: OPLS parameters for mass in Da and charge in e.

Atom type	Mass	Charge		
	OPLS[4–6]	OPLS-AA[7]	OPLS-AA-D,[8] OPLS-S,[9] OPLS-G[9]	OPLS-AA-N[4–6]
C	12.01100	0.500	0.142	0.95929
O	15.99940	-0.500	-0.390	-0.51455
N	14.00670	-0.760	-0.542	-1.30043
H	1.00800	0.380	0.333	0.53904

Table 6: The OPLS Lennard-Jones parameters used for urea. ε in kcal mol^{-1} and σ in Å.

Atom type	OPLS-AA,[7] OPLS-AA-D,[8] OPLS-S,[9] OPLS-G[9]		OPLS-AA-N[4–6]	
	ε	σ	ε	σ
C	0.105	3.750	0.070	3.550
O	0.210	2.960	0.210	2.960
N	0.170	3.250	0.170	3.250
H	0.000	0.000	0.000	0.000

Table 7: The bond and angle parameters used with OPLS for urea. K_r in kcal mol^{-1} Å^{-4}, r_0 in Å, K_θ in kcal mol^{-1} rad^{-2} and θ_0 in °. The K_r and K_θ values have been multiplied by $\frac{1}{4}$ and $\frac{1}{2}$ respectively, to match the LAMMPS formatting convention. * The N - C - N bond is not used in OPLS-AA.

	OPLS-G,[9,10] OPLS-S[9,10]		OPLS-AA,[7,11] OPLS-AA-N,[4–6] OPLS-AA-D[4–6]	
Bonds	K_r	r_0	K_r	r_0
C - O	78.3	1.2650	570.0	1.2290
C - N	61.5	1.3500	490.0	1.3350
N - H	111.7	1.0000	434.0	1.0100
Angles	K_θ	θ_0	K_θ	θ_0
N - C - O	82.5	121.4	80.0	122.9
N - C - N*	76.0	117.2	70.0	114.2
C - N - H	46.6	120.0	35.0	119.8
H - N - H	53.2	120.0	35.0	120.0

Table 8: The dihedral and improper dihedral parameters used with OPLS for urea. V in kcal mol^{-1}, K_ξ in kcal mol^{-1} rad^{-2} and n and d are unit less.

	OPLS-AA,[7] OPLS-AA-N,[4–6] OPLS-AA-D[4–6]			
Dihedrals	V_1	V_2	V_3	V_4
H - N - C - N	0.000	4.900	0.000	0.000
H - N - C - O	0.000	4.900	0.000	0.000

Improper dihedrals		K_ξ	n	d
O - C - N - N		10.5	2	-1
C - N - H - H		2.5	2	-1

Table 9: The dihedral and improper dihedral parameters used with OPLS for urea. K_ϕ in kcal mol^{-1}, d and n are unit less, K_ξ in kcal mol^{-1} rad^{-2} and ξ_0 in °. The K_ξ value has been multiplied by $\frac{1}{2}$ and 180° has been added to ξ_0 to match the LAMMPS formatting convention.

	OPLS-S[9]			OPLS-G[10]		
Dihedrals	K_ϕ	d	n	K_ϕ	d	n
H - N - C - O	9.99	-1	2	8.01	-1	2

Improper dihedrals	K_ξ		ξ_0	K_ξ		ξ_0
O - C - N - N	20.0		180	20.0		180
C - N - H - H	20.0		180	20.0		180

A.4 SPC/E water parameters

Table 10: SPC/E parameters for mass, charge and Lennard-Jones parameters for water. Mass in Da, charge in e, ε in kcal mol^{-1} and σ in Å.

Atom type	Mass	Charge[12,13]	ϵ[12,13]	σ[12,13]
O	16.00	-0.8476	0.1553	3.1656
H	1.008	0.4238	0.0000	0.0000

Table 11: SPC/E bond and angle parameters for water.[12,13] K_r in kcal mol^{-1} Å^{-2}, r_0 in Å, K_θ in kcal mol^{-1} rad^{-2} and r_0 in °.

Bond	K_r	r_0
O - H	1000.0	1.000

Angle	K_θ	r_0
H - O - H	100.0	109.47

B Tabulated results

B.1 Crystal lattice parameters

Table 12: Crystal lattice parameters after minimisation of the experimental form I crystal structure.

Force field	a (Å)	b (Å)	c (Å)	ρ (g cm^{-3})
GAFF1	5.324	5.324	4.820	1.460
GAFF2	5.321	5.321	4.774	1.476
GAFF-D1	5.221	5.221	4.810	1.521
GAFF-D3	5.328	5.328	4.811	1.460
OPLS-AA	5.412	5.412	4.795	1.420
OPLS-AA-N	5.350	5.350	4.830	1.442
OPLS-AA-D	5.415	5.415	4.786	1.421
OPLS-S	5.493	5.493	4.785	1.382
OPLS-G	5.490	5.490	4.775	1.386

Table 13: Crystal lattice parameters after minimisation of the experimental form IV crystal structure.

Force field	a (Å)	b (Å)	c (Å)	ρ (g cm^{-3})
GAFF1	3.505	7.523	4.847	1.561
GAFF2	3.499	7.102	4.796	1.673
GAFF-D1	3.512	7.052	4.813	1.673
GAFF-D3	3.507	7.454	4.817	1.584
OPLS-AA	3.576	7.614	4.883	1.500
OPLS-AA-N	3.646	7.181	5.222	1.459
OPLS-AA-D	3.698	7.569	4.882	1.460
OPLS-S	3.660	7.688	4.886	1.451
OPLS-G	3.649	7.696	4.888	1.453

Table 14: Crystal lattice parameters from the NPT simulations starting in form I. The mean and standard deviation are calculated with a sampling frequency of 0.1 ns.

Force field	a (Å)		b (Å)		c (Å)		ρ (g cm^{-3})	
	Mean	St. dev.	Mean	St. dev.	Mean	St. dev.	Mean	St. dev.
GAFF1	5.451	0.230	5.447	0.239	4.715	0.011	1.427	0.007
GAFF2	5.405	0.237	5.409	0.238	4.685	0.010	1.459	0.007
GAFF-D1	5.253	0.030	5.252	0.030	4.605	0.012	1.570	0.007
GAFF-D3	5.398	0.082	5.400	0.082	4.691	0.011	1.459	0.006
OPLS-AA	5.409	0.123	5.408	0.125	4.681	0.011	1.457	0.006
OPLS-AA-N	5.091	0.042	5.680	0.031	4.405	0.033	1.566	0.009
OPLS-AA-D	4.691	0.069	6.317	0.078	4.673	0.011	1.441	0.007
OPLS-S	4.830	0.082	6.207	0.090	4.701	0.010	1.415	0.007
OPLS-G	4.893	0.102	6.141	0.108	4.699	0.010	1.413	0.008

Table 15: Crystal lattice parameters from the NPT simulations starting in form IV. The mean and standard deviation are calculated with a sampling frequency of 0.1 ns.

Force field	a (Å)		b (Å)		c (Å)		ρ (g cm^{-3})	
	Mean	St. dev.	Mean	St. dev.	Mean	St. dev.	Mean	St. dev.
GAFF1	3.779	0.033	7.174	0.039	4.735	0.010	1.554	0.009
GAFF2	3.740	0.034	7.104	0.037	4.705	0.011	1.596	0.010
GAFF-D1	3.675	0.024	7.646	0.042	4.385	0.019	1.619	0.008
GAFF-D3	3.814	0.037	7.080	0.049	4.689	0.013	1.575	0.009
OPLS-AA	5.402	0.146	5.416	0.152	4.682	0.012	1.457	0.006
OPLS-AA-N	3.835	0.016	6.929	0.023	4.591	0.030	1.635	0.009
OPLS-AA-D	4.688	0.070	6.322	0.081	4.673	0.012	1.440	0.008
OPLS-S	4.837	0.087	6.200	0.100	4.701	0.012	1.415	0.007
OPLS-G	4.885	0.103	6.149	0.112	4.700	0.012	1.413	0.008

B.2 Crystal potential energy

Table 16: Crystal potential energy in kJ mol^{-1} after minimisation of the experimental form IV crystal structure.

Force field	Starting in form I	Starting in form IV	Energy difference
GAFF1	-543.20	-540.86	-2.34
GAFF2	-541.36	-543.23	1.87
GAFF-D1	-1172.58	-1171.01	-1.57
GAFF-D3	-807.82	-804.61	-3.21
OPLS-AA	-332.00	-319.02	-12.98
OPLS-AA-N	-572.47	-560.49	-11.98
OPLS-AA-D	-215.92	-205.53	-10.39
OPLS-S	-213.68	-201.32	-12.36
OPLS-G	-213.96	-200.80	-13.16

Table 17: Crystal potential energy in kJ mol^{-1} for the NPT simulations. The mean value and standard deviation are calculated with a sampling frequency of 0.1 ns.

Force field	Starting in form I		Starting in form IV		Energy difference	
	Mean	St. dev.	Mean	St. dev.	Mean	St. dev.
GAFF1	-510.26	1.22	-511.52	1.12	1.26	2.34
GAFF2	-507.22	1.26	-508.81	1.28	1.59	2.54
GAFF-D1	-1143.52	1.14	-1148.22	0.97	4.70	2.10
GAFF-D3	-775.11	1.22	-775.90	1.12	0.79	2.34
OPLS-AA	-301.24	1.16	-301.22	0.85	-0.02	2.00
OPLS-AA-N	-558.30	0.90	-577.03	0.87	18.73	1.78
OPLS-AA-D	-184.46	1.18	-184.40	0.92	-0.06	2.10
OPLS-S	-181.97	1.21	-182.01	0.87	0.04	2.08
OPLS-G	-182.00	1.29	-181.96	0.88	-0.04	2.08

B.3 Crystal cohesive energy

Table 18: Crystal cohesive energy in kJ mol^{-1} for the NPT simulations. The mean value and standard error are calculated with a sampling frequency of 0.1 ns.

Force field	Starting in form I		Starting in form I		Energy difference	
	Mean	St. err.	Mean	St. err.	Mean	St. err.
GAFF1	-81.3	0.3	-82.6	0.3	1.3	0.6
GAFF2	-80.0	0.4	-81.6	0.4	1.6	0.8
GAFF-D1	-113.5	0.4	-118.2	0.4	4.7	0.8
GAFF-D3	-91.9	0.4	-92.7	0.4	0.8	0.8
OPLS-AA	-87.5	0.3	-87.5	0.3	0.0	0.7
OPLS-AA-N	-98.8	0.3	-117.5	0.3	18.7	0.6
OPLS-AA-D	-83.7	0.4	-83.7	0.4	0.0	0.8
OPLS-S	-79.3	0.3	-79.4	0.3	0.1	0.6
OPLS-G	-80.2	0.3	-80.2	0.3	0.1	0.6

B.4 Solution density

Table 19: Solution density mean and standard deviation, calculated with a sampling frequency of 0.1 ns.

Concentration		Density (g cm^{-3})					
Urea molecules	Mass %	GAFF1		GAFF2		GAFF-D1	
		Mean	St. dev.	Mean	St. dev.	Mean	St. dev.
0	0.00	0.999	0.008	0.999	0.008	0.999	0.008
10	3.23	1.008	0.008	1.008	0.008	1.012	0.008
50	14.29	1.041	0.007	1.044	0.008	1.063	0.008
100	25.00	1.076	0.007	1.080	0.007	1.115	0.007
150	33.34	1.104	0.007	1.110	0.008	1.158	0.007
200	40.00	1.128	0.007	1.136	0.007	1.195	0.006
300	50.00	1.167	0.006	1.176	0.007	1.251	0.005
400	57.15	1.196	0.006	1.206	0.005	1.293	0.005
500	62.50	1.218	0.005	1.231	0.006	1.325	0.005
600	66.67	1.238	0.005	1.251	0.006	1.351	0.005
1000	76.93	1.285	0.004	1.301	0.004	1.417	0.004

Table 20: Solution density mean and standard deviation, calculated with a sampling frequency of 0.1 ns.

Concentration		Density (g cm^{-3})					
Urea molecules	Mass %	GAFF-D3		OPLS-AA		OPLS-AA-N	
		Mean	St. dev.	Mean	St. dev.	Mean	St. dev.
0	0.00	0.999	0.008	0.999	0.008	0.999	0.008
10	3.23	1.009	0.008	1.009	0.008	1.014	0.008
50	14.29	1.048	0.008	1.047	0.008	1.073	0.007
100	25.00	1.087	0.007	1.086	0.007	1.129	0.007
150	33.34	1.120	0.007	1.118	0.006	1.177	0.007
200	40.00	1.149	0.007	1.144	0.006	1.216	0.007
300	50.00	1.192	0.007	1.184	0.005	1.274	0.006
400	57.15	1.225	0.006	1.215	0.006	1.321	0.005
500	62.50	1.250	0.006	1.238	0.005	1.356	0.005
600	66.67	1.271	0.005	1.256	0.005	1.384	0.004
1000	76.93	1.323	0.004	1.303	0.004	1.453	0.004

Table 21: Solution density mean and standard deviation, calculated with a sampling frequency of 0.1 ns.

Concentration		Density (g cm^{-3})					
Urea molecules	Mass %	OPLS-AA-D		OPLS-S		OPLS-G	
		Mean	St. dev.	Mean	St. dev.	Mean	St. dev.
0	0.00	0.999	0.008	0.999	0.008	0.999	0.008
10	3.23	1.009	0.008	1.007	0.008	1.007	0.007
50	14.29	1.045	0.008	1.041	0.008	1.042	0.008
100	25.00	1.082	0.008	1.077	0.007	1.076	0.007
150	33.33	1.112	0.007	1.105	0.007	1.105	0.007
200	40.00	1.137	0.007	1.129	0.006	1.129	0.007
300	50.00	1.175	0.006	1.165	0.006	1.165	0.006
400	57.15	1.206	0.005	1.193	0.005	1.193	0.005
500	62.50	1.228	0.005	1.215	0.006	1.214	0.005
600	66.67	1.246	0.005	1.232	0.005	1.231	0.005
1000	76.93	1.292	0.004	1.273	0.004	1.274	0.004

B.5 Solution diffusion coefficients

Table 22: Solution diffusion coefficient, with standard error, for the GAFF force fields. The gradient and associated standard error of the MSD was calculated over the first 10 ns of the MSD. Trajectory data was used every 0.01 ns for calculating the multi-time-origin MSD.

Concentration		Diffusion coefficient ($\times 10^{-9}$ m^2 s^{-1})					
Urea molecules	Mass %	GAFF1		GAFF2		GAFF-D1	
		Mean	St. err.	Mean	St. err.	Mean	St. err.
10	3.23	1.9834	0.0028	1.0856	0.0029	1.3211	0.0021
50	14.29	1.7137	0.0018	1.4226	0.0005	0.9952	0.0013
100	25.00	1.2271	0.0007	1.2938	0.0004	0.8596	0.0010
150	33.34	1.1464	0.0005	1.1569	0.0004	0.7770	0.0004
200	40.00	1.0678	0.0008	1.1146	0.0002	0.6362	0.0002
300	50.00	0.8090	0.0001	0.8867	0.0004	0.4812	0.0003
400	57.15	0.6790	0.0002	0.8568	0.0002	0.3583	0.0000
500	62.50	0.6055	0.0001	0.7220	0.0002	0.3004	0.0000
600	66.67	0.5482	0.0001	0.6492	0.0002	0.2446	0.0000
1000	76.93	0.3806	0.0000	0.4602	0.0001	0.1227	0.0000

Table 23: Solution diffusion coefficient, with standard error, for the GAFF force fields. The gradient and associated standard error of the MSD was calculated over the first 10 ns of the MSD. Trajectory data was used every 0.01 ns for calculating the multi-time-origin MSD.

Concentration		Diffusion coefficient ($\times 10^{-9}$ m^2 s^{-1})					
Urea molecules	Mass %	GAFF-D3		OPLS-AA		OPLS-AA-N	
		Mean	St. err.	Mean	St. err.	Mean	St. err.
10	3.23	1.4841	0.0017	1.5908	0.0034	0.6659	0.0033
50	14.29	1.3152	0.0004	1.4147	0.0006	0.5662	0.0001
100	25.00	1.2854	0.0005	1.3645	0.0009	0.3257	0.0002
150	33.34	1.1776	0.0006	1.2464	0.0003	0.2409	0.0001
200	40.00	1.1197	0.0008	1.0898	0.0004	0.1517	0.0000
300	50.00	0.8446	0.0005	0.8198	0.0004	0.0641	0.0000
400	57.15	0.7509	0.0001	0.7266	0.0001	0.0287	0.0000
500	62.50	0.6460	0.0001	0.6370	0.0001	0.0139	0.0000
600	66.67	0.5798	0.0001	0.5970	0.0001	0.0052	0.0000
1000	76.93	0.4044	0.0001	0.3924	0.0001	0.0005	0.0000

Table 24: Solution diffusion coefficient, with standard error, for the GAFF force fields. The gradient and associated standard error of the MSD was calculated over the first 10 ns of the MSD. Trajectory data was used every 0.01 ns for calculating the multi-time-origin MSD.

Concentration		Diffusion coefficient ($\times 10^{-9}$ m^2 s^{-1})					
Urea molecules	Mass %	OPLS-AA-D		OPLS-S		OPLS-G	
		Mean	St. err.	Mean	St. err.	Mean	St. err.
10	3.23	1.9129	0.0024	2.3257	0.0059	1.2183	0.0039
50	14.29	1.7211	0.0009	1.2912	0.0013	1.4087	0.0023
100	25.00	1.4297	0.0011	1.3815	0.0008	1.4043	0.0014
150	33.34	1.2541	0.0004	1.3348	0.0004	1.3397	0.0003
200	40.00	1.1676	0.0001	1.2838	0.0009	1.1421	0.0004
300	50.00	0.9507	0.0005	0.9901	0.0002	0.9408	0.0002
400	57.15	0.8224	0.0002	0.8760	0.0002	0.8471	0.0003
500	62.50	0.7665	0.0003	0.7247	0.0002	0.7873	0.0001
600	66.67	0.6778	0.0001	0.6922	0.0003	0.7174	0.0002
1000	76.93	0.4859	0.0001	0.4789	0.0000	0.4827	0.0001

C Further Radial Distribution Functions

The O–H_W and O–N RDFs have been discussed in the main paper, here we also provide RDFs and discussion for the following interactions H–O_W, O–O_W, N–H_W, N–O_W and C–O_W. The RDFs have been calculated up to 9.0 Å, at which point the curves are tailing off to a value of 1.0 for all the different RDF interactions and force fields studied. The only exception is the O–N RDFs for the dilute solutions, which is also tailing off but more slowly.

C.1 H–O_W

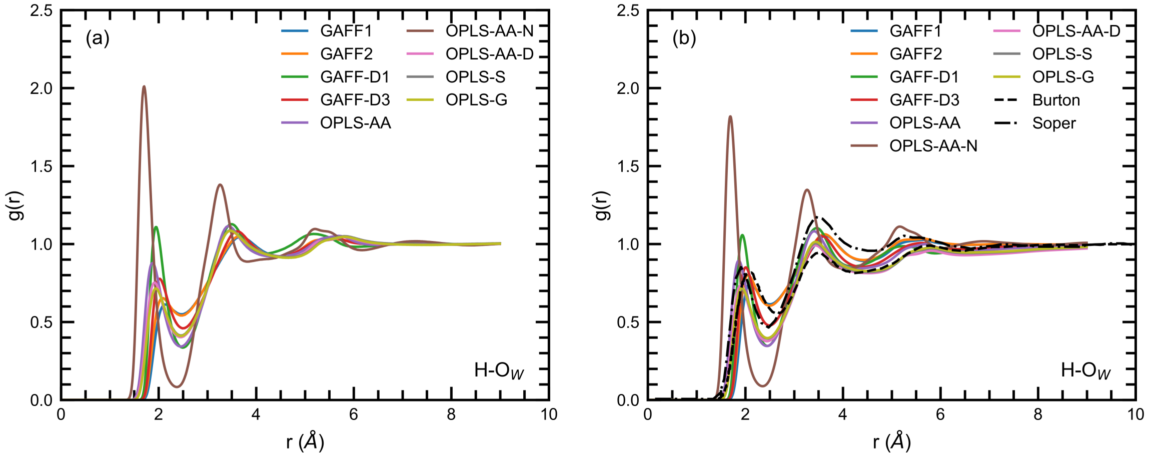

Figure 1: H–O_W RDFs for (a) the dilute solutions and (b) the concentrated solutions. Literature data is taken from Burton et al.[14] and Soper et al.[15].

The H–O_W RDFs in Figure 1 are similar for all the force fields and concentrations, except that OPLS-AA-N has greatly extended peaks and troughs, which also appear earlier. Like with O–H_W there is the first peak at < 2 Å, indicating that strong hydrogen bonding is present. There are also weaker second and third peaks appearing at 3.5 Å and > 5.0 Å respectively. The H–O_W RDFs are similar to those obtained by Burton et al.[14] and Soper et al.[15], and also from Ishida et al.[16] and Duffy et al.[8] but these had separate RDFs for the urea H molecules in both syn and anti arrangements, which were not shown for clarity. The RDFs of Duffy et al.[8] indicate that the broader third peak is made up of two pairs of smaller peaks from these (syn peak, anti peak, syn peak, anti peak).

C.2 O–O_W

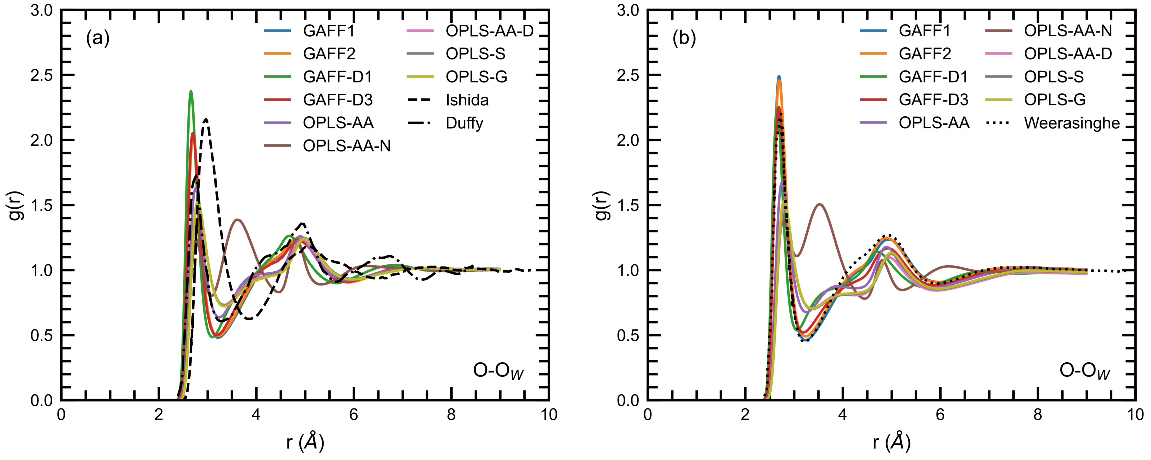

Figure 2: O–O_W RDFs for (a) the dilute solutions and (b) the concentrated solutions. Literature data is taken from Ishida et al.[16], Duffy et al.[8], and Weerasinghe and Smith[17].

For the O–O_W RDFs, shown in in Figure 2, there is a strong first peak at > 2.5 Å followed by broader second and third peaks for all the force fields apart from OPLS-AA-N. For the remaining OPLS force fields a small second peak occurs at > 3.5 Å, which plateaus before the third peak appears at 5 Å. For the GAFF force fields a slow rise leads to the second peak which appears later at > 4.0 Å, this peak is higher that that for the OPLS force fields. This later, taller second peak runs into the third peak at 5.0 Å. This shape is similar to that obtained by Duffy et al.[8] and Weerasinghe and Smith[17]. These peaks are very similar for both the dilute and concentrated systems. The overlapping second and third peaks, could be a combination of water molecules not directly bonded to the O on the urea molecule and water molecules hydrogen bonded to the other parts of the urea molecule. OPLS-AA-N has first and third peaks very similar to those of the other OPLS force fields. However, the second OPLS-AA-N peak is much stronger and more well defined than those of the other force fields, and it Speaks earlier at 3.5 Å. This is not just due to the O–O_W partial charges (and also those of O–H_W), since the charge differences for OPLS-AA-N lies between those of the other OPLS force fields and GAFF force fields.

C.3 N–H_W

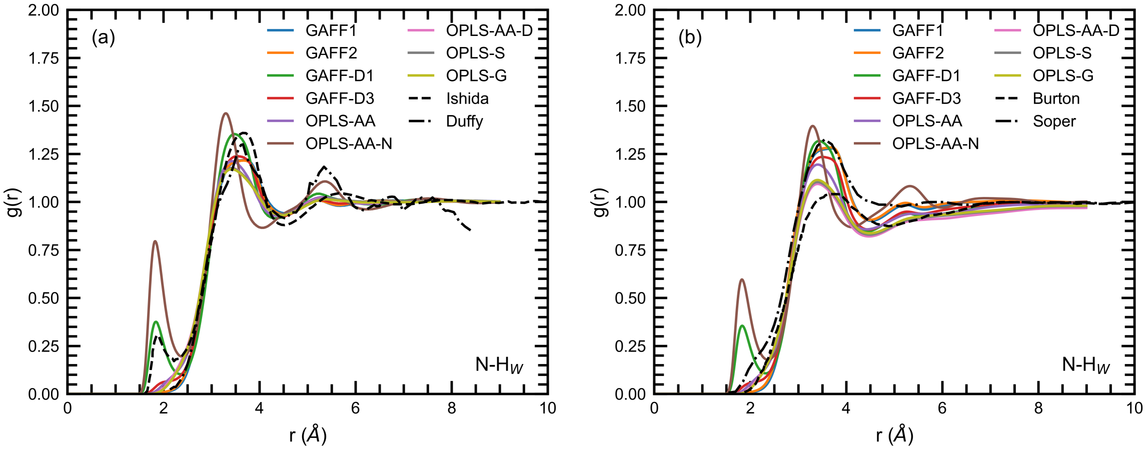

Figure 3: N–H_W RDFs for (a) the dilute solutions and (b) the concentrated solutions. Literature data is taken from Ishida et al.[16], Duffy et al.[8], Burton et al.[14], and Soper et al.[15]

The N–H_W RDF, in Figure 3, has an extra peak at < 2 Å for GAFF-D1 and OPLS-AA-N, and which is very weak for GAFF-D3 and absent from the other force fields. This matches a very clear peak in Ishida et al.[16], whilst there is only a small bump in Soper et al.[15] and no peak in Duffy et al.[8] and Burton et al.[14]. This indicates that there are only strong N$\cdots$$H_W$ hydrogen bonds presents in GAFF-D1 and OPLS-AA-N, with a few present in GAFF-D3. These additional hydrogen bonds can be related to the higher density of these force fields. The first main peak appears at > 3.0 Å, which also appears in all the reference RDFs. There is a second peak at > 5.0 Å, this not noticeable in the higher concentrations of OPLS or reference solutions, with the exception of OPLS-AA-N where this is enhanced in the higher concentrations.

C.4 N–O_W

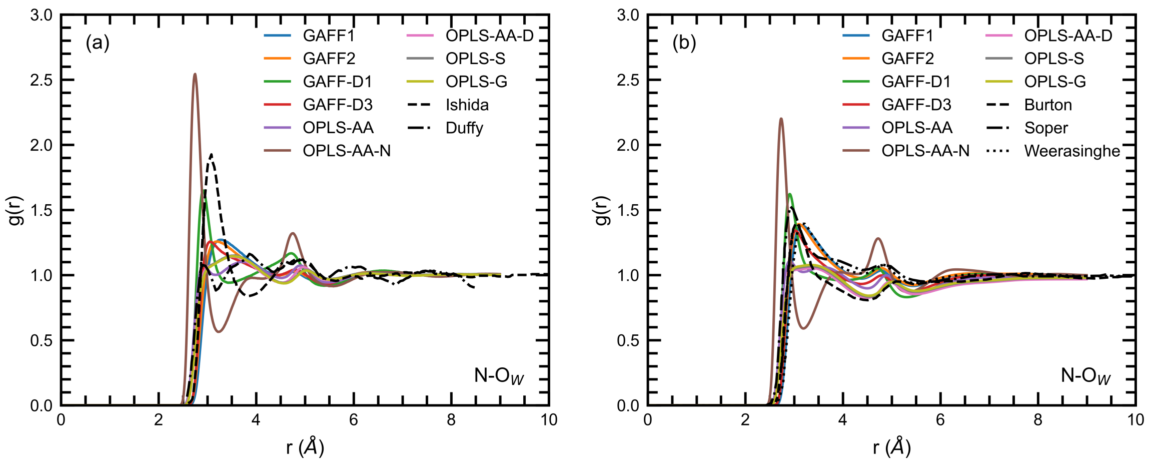

Figure 4: N–O_W RDFs for (a) the dilute solutions and (b) the concentrated solutions. Literature data is taken from Ishida et al.[16], Duffy et al.[8], Burton et al.[14], Soper et al.[15], and Weerasinghe and Smith[17].

Interestingly, the charge on the N atom is more negative than that of the O_W atom for GAFF-D1, GAFF-D3 and OPLS-AA-N, but this is the opposite for the remaining force fields. Despite this there is no significant difference between the N–O_W RDF of GAFF-D3 (for which the N and OW charges are very similar) compared to GAFF1 and GAFF2, as shown in Figure 4. The OPLS RDF peaks are generally lower and shifted to the right compared to those of the GAFF force fields. Again, OPLS-AA-N provides the exception, with significantly taller first and second peaks, and the first peak further to the left than that of the other force fields. The GAFF-D1 RDF sits somewhere between that of OPLS-AA-N and the other GAFF force fields, getting more like the GAFF RDFs at higher concentrations, where the water-to-urea ration decreases. In the dilute solutions neither Ishida et al.[16] or Duffy et al.[8] match the first peaks obtained here. However, at the higher concentrations, the Weerasinghe and Smith[17] curve matches the GAFF RDFs well, the Burton et al.[14] curve has a taller first peak but otherwise is similar to the OPLS RDFs and Soper et al.[15] has similarities to GAFF-D1.

C.5 C–O_W

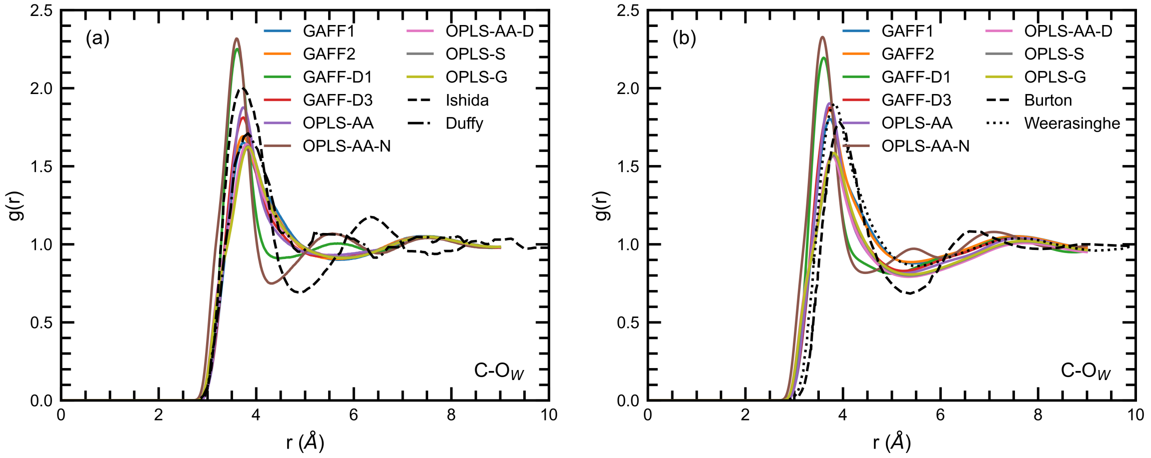

Figure 5: C–O_W RDFs for (a) the dilute solutions and (b) the concentrated solutions. Literature data is taken from Ishida et al. [16], Duffy et al. [8], Burton et al. [14], and Weerasinghe and Smith [17].

The C–O_W RDFs in Figure 5 have one main peak at $>$ 3.5 Å and a lower broad peak at $\geq$ 7.5 Å as it is levelling off, the RDFs are similar for both the dilute and concentrated solutions. OPLS-AA-N and GAFF-D1 have significantly taller first peaks than the other force fields. In the dilute systems OPLS-AA-N and GAFF-D1 both have an additional peak at $\sim$ 5.5 Å, this also occurred for Duffy et al. [8] this is also present at the higher concentrations for OPLS-AA-N only. The RDFs of Weerasinghe and Smith [17] matches well, so does Burton et al. [14] although the second peak is shifted slightly earlier and this is significantly earlier in Ishida et al. [16].

References

(1) Case, D. A. et al. AmberTools21. University of California, San Francisco, 2021.

(2) Özpınar, G. A.; Peukert, W.; Clark, T. An Improved Generalized AMBER Force Field (GAFF) for Urea. *J. Mol. Model.* **2010**, *16*, 1427–1440.

(3) Wang, J.; Wolf, R. M.; Caldwell, J. W.; Kollman, P. A.; Case, D. A. Development and Testing of a General Amber Force Field. *J. Comput. Chem.* **2004**, *25*, 1157–1174.

(4) Dodda, L. S.; Vilseck, J. Z.; Tirado-Rives, J.; Jorgensen, W. L. 1.14*CM1A-LBCC: Localized Bond-Charge Corrected CM1A Charges for Condensed-Phase Simulations. *J. Phys. Chem. B* **2017**, *121*, 3864–3870.

(5) Dodda, L. S.; Cabeza de Vaca, I.; Tirado-Rives, J.; Jorgensen, W. L. LigParGen Web Server: An Automatic OPLS-AA Parameter Generator for Organic Ligands. *Nucleic Acids Research* **2017**, *45*, W331–W336.

(6) Jorgensen, W. L.; Tirado-Rives, J. Potential Energy Functions for Atomic-Level Simulations of Water and Organic and Biomolecular Systems. *Proc. Natl. Acad. Sci. U.S.A.* **2005**, *102*, 6665–6670.

(7) Jorgensen, W. L.; Maxwell, D. S.; Tirado-Rives, J. Development and Testing of the OPLS All-Atom Force Field on Conformational Energetics and Properties of Organic Liquids. *J. Am. Chem. Soc.* **1996**, *118*, 11225–11236.

(8) Duffy, E. M.; Severance, D. L.; Jorgensen, W. L. Urea: Potential Functions, Log P, and Free Energy of Hydration. *Isr. J. Chem.* **1993**, *33*, 323–330.

(9) Smith, L. J.; Berendsen, H. J. C.; van Gunsteren, W. F. Computer Simulation of Urea-Water Mixtures: A Test of Force Field Parameters for Use in Biomolecular Simulation. *J. Phys. Chem. B* **2004**, *108*, 1065–1071.

(10) Oostenbrink, C.; Villa, A.; Mark, A. E.; Van Gunsteren, W. F. A Biomolecular Force Field Based on the Free Enthalpy of Hydration and Solvation: The GROMOS Force-Field Parameter Sets 53A5 and 53A6. *J. Comput. Chem.* **2004**, *25*, 1656–1676.

(11) Weiner, S. J.; Kollman, P. A.; Nguyen, D. T.; Case, D. A. An All Atom Force Field for Simulations of Proteins and Nucleic Acids: An All Atom Force Field. *J. Comput. Chem.* **1986**, *7*, 230–252.

(12) Berendsen, H. J. C.; Grigera, J. R.; Straatsma, T. P. The Missing Term in Effective Pair Potentials. *J. Phys. Chem.* **1987**, *91*, 6269–6271.

(13) Mark, P.; Nilsson, L. Structure and Dynamics of the TIP3P, SPC, and SPC/E Water Models at 298 K. *J. Phys. Chem. A* **2001**, *105*, 9954–9960.

(14) Burton, R. C.; Ferrari, E. S.; Davey, R. J.; Hopwood, J.; Quayle, M. J.; Finney, J. L.; Bowron, D. T. The Structure of a Supersaturated Solution: A Neutron Scattering Study of Aqueous Urea. *Cryst. Growth Des.* **2008**, *8*, 1559–1565.

(15) Soper, A.; Castner, E.; Luzar, A. Impact of Urea on Water Structure: A Clue to Its Properties as a Denaturant? *Biophysical Chemistry* **2003**, *105*, 649–666.

(16) Ishida, T.; Rossky, P. J.; Castner, E. W. A Theoretical Investigation of the Shape and Hydration Properties of Aqueous Urea: Evidence for Nonplanar Urea Geometry. *J. Phys. Chem. B* **2004**, *108*, 17583–17590.

(17) Weerasinghe, S.; Smith, P. E. A Kirkwood-Buff Derived Force Field for Mixtures of Urea and Water. *J. Phys. Chem. B* **2003**, *107*, 3891–3898.
